# Targeting the glucocorticoid receptor signature gene Mono Amine Oxidase-A enhances the efficacy of chemo- and anti-androgen therapy in advanced prostate cancer

**DOI:** 10.1038/s41388-021-01754-0

**Published:** 2021-04-01

**Authors:** Martin Puhr, Andrea Eigentler, Florian Handle, Hubert Hackl, Christian Ploner, Isabel Heidegger, Georg Schaefer, Maximilian P. Brandt, Julia Hoefer, Gabri Van der Pluijm, Helmut Klocker

**Affiliations:** 1 Department of Urology, Medical University of Innsbruck, Innsbruck, Austria; 2 Institute of Bioinformatics, Biocenter, Medical University of Innsbruck, Innsbruck, Austria; 3 Department of Plastic, Reconstructive and Aesthetic Surgery Innsbruck, Medical University of Innsbruck, Innsbruck, Austria; 4 Department of Pathology, Medical University of Innsbruck, Innsbruck, Austria; 5 Department of Urology, University Medical Center, Johannes Gutenberg University, Mainz, Germany; 6 Department of Urology, Leiden University Medical Center, Leiden, The Netherlands

**Keywords:** Prostate cancer, Hormone receptors

## Abstract

Despite increasing options for treatment of castration-resistant prostate cancer, development of drug resistance is inevitable. The glucocorticoid receptor (GR) is a prime suspect for acquired therapy resistance, as prostate cancer (PCa) cells are able to increase GR signaling during anti-androgen therapy and thereby circumvent androgen receptor (AR)-blockade and cell death. As standard AR-directed therapies fail to block the GR and GR inhibitors might result in intolerable side effects, the identification of GR signature genes, which are better suited for a targeted approach, is of clinical importance. Therefore, the specific epithelial and stromal GR signature was determined in cancer-associated fibroblasts as well as in abiraterone and enzalutamide-resistant cells after glucocorticoid (GC) treatment. Microarray and ChIP analysis identified MAO-A as a directly up-regulated mutual epithelial and stromal GR target, which is induced after GC treatment and during PCa progression. Elevated MAO-A levels were confirmed in in vitro cell models, in primary tissue cultures after GC treatment, and in patients after neoadjuvant chemotherapy with GCs. MAO-A expression correlates with GR/AR activity as well as with a reduced progression-free survival. Pharmacological MAO-A inhibition combined with 2^nd^ generation AR signaling inhibitors or chemotherapeutics results in impaired growth of androgen-dependent, androgen-independent, and long-term anti-androgen-treated cells. In summary, these findings demonstrate that targeting MAO-A represents an innovative therapeutic strategy to synergistically block GR and AR dependent PCa cell growth and thereby overcome therapy resistance.

## Introduction

Novel therapeutic regimen have expanded the limited options for treatment of advanced prostate cancer (PCa) including 2^nd^ generation AR signaling inhibitors [1–4], or taxane based chemotherapeutics [5, 6]. However, treatment of metastatic PCa remains palliative due to the inevitable occurrence of acquired therapy-resistance [7–9], claiming the need for new targeting strategies in order to achieve a personalized treatment approach.

Aside from androgen receptor (AR) modifications, which have already been linked to therapy failure, the glucocorticoid receptor (GR) has also been identified as a suspect for acquired therapy resistance. Enhanced GR expression was observed in enzalutamide-resistant tumors in vivo as well as in metastatic biopsies from enzalutamide-treated PCa patients [10]. In line with these results, our group observed significantly elevated GR expression in multiple long-term abiraterone and enzalutamide treated PCa cells, and demonstrated a correlation between enhanced epithelial GR expression and early biochemical relapse. Increased GR expression was also present in patient tissue samples after neoadjuvant chemotherapy, and in docetaxel resistant PCa cells [11]. Concordantly, administration of the GR inhibitor RU486 resulted in elevated apoptosis, suggesting a beneficial effect of GR blockade together with ADT or chemotherapy. However, until now, research has solely focused on epithelial GR expression and pharmacological GR inhibition, and neglected the consequences of altered epithelial and stromal GR signaling. Downstream analysis is particularly important due to the fact that glucocorticoids (GCs), which are the primary ligands of the GR, are routinely administered in clinical practice to minimize therapy related side effects [5, 12, 13]. Therefore, systemic GR inhibition or regimens excluding GCs are, in most cases, not clinically feasible. Instead, there is an unmet medical need to identify elevated GR downstream effectors, which may be more suitable as clinically modifiable targets.

In the present study, we have investigated the altered epithelial and stromal GR signature in PCa cells after GC treatment and identified monoamine oxidase A (MAO-A) as a mutual directly up-regulated epithelial and stromal GR target. MAO-A is a mitochondrial membrane-bound oxidoreductase that catalyzes the degradation of biogenic and xenobiotic amines [14, 15]. Elevated amine metabolism is linked to altered cell growth and to reactive oxygen species (ROS), which are known to cause oxidative damage with implications for aging and cancer. Concerning PCa, induced MAO-A expression is correlated with tumor progression and therapy resistance [16, 17]. Nevertheless, the impact of GC medication on MAO-A expression has not been investigated so far.

Here, we report significantly elevated MAO-A levels in diverse cell models, representing different disease stages and in primary PCa ex vivo tissue cultures after GC treatment, as well as in radical prostatectomy (RPE) specimens after neoadjuvant chemotherapy in combination with GCs. Furthermore, we identify MAO-A targeting in combination with 2^nd^ generation AR signaling inhibitors and chemotherapeutics as a promising novel treatment option for advanced PCa.

## Results

### Identification of the epithelial and stromal GR signature

**Fig. 1:**
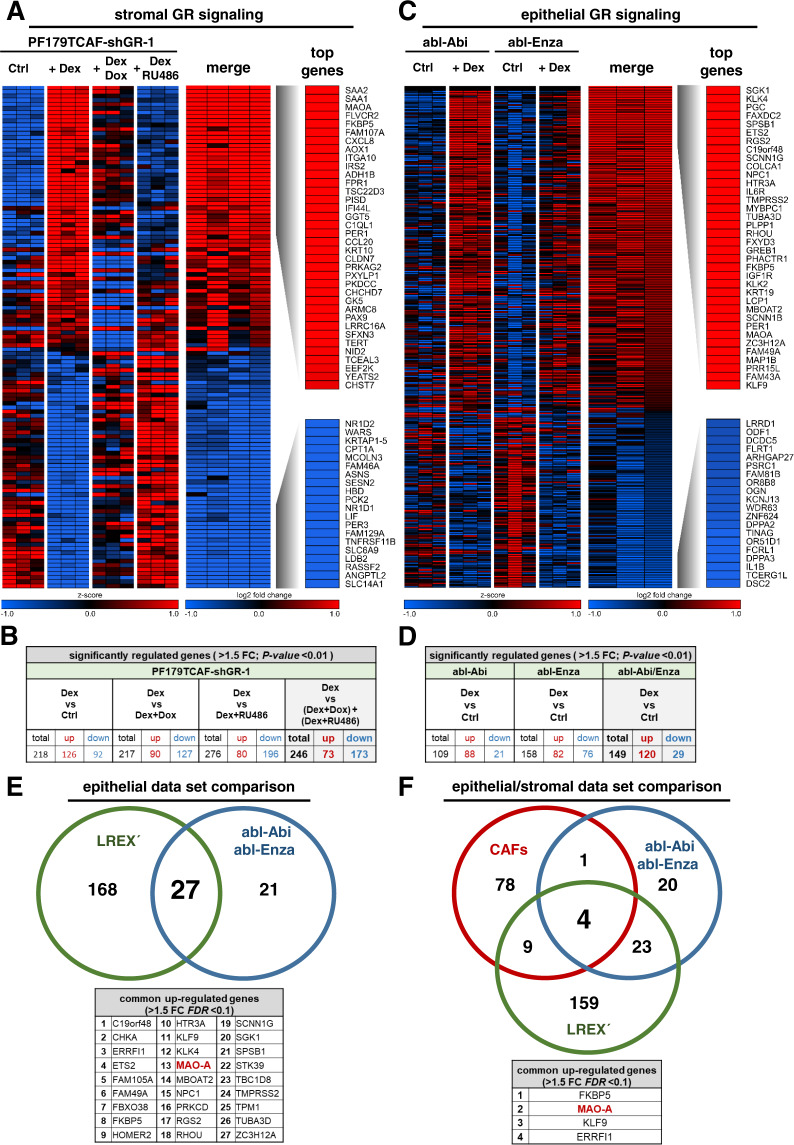
Identification of the stromal and epithelial GR target gene signature after GC treatment. **A** Heat map of top 36 significantly up-regulated and top 20 significantly down-regulated genes of stromal PF179TCAF-shGR-1 cells. **B** Significantly altered GR target genes of PF179TCAF-shGR-1 cells, after control DMSO treatment as well as after 100 nM Dex, 100 nM Dex + 1 µg/ml Dox, and 100 nM Dex and 12 µM RU486 treatment for 24 h. **C** Heat map of top 36 significantly up-regulated and top 20 significantly down-regulated genes of epithelial abl-Enza and abl-Abi cells. **D** Significantly altered GR target genes of abl-Enza and abl-Abi cells, after control DMSO treatment as well as after 100 nM Dex treatment for 24 h. **E** Identification of 27 mutual significantly up-regulated GR target genes between the LREX´ and abl-Enza/Abi datasets. **F** Identification of four mutual significantly up-regulated stromal and epithelial GR target genes within the CAF, abl-Enza/Abi and LREX´ datasets.

In order to identify GR target genes that might be suitable for therapeutic targeting of the GR network in PCa we first assessed the specific epithelial and stromal GR signature. For the stromal GR signature we utilized the PF179TCAF cell model with stable integration of a doxycycline (Dox) inducible short hairpin RNA (shRNA) vector against human GR (PF179TCAF-shGR-1). A microarray comparison analysis after GR activation with dexamethasone (Dex), GR knockdown (Dox), as well as pharmacological GR inhibition (RU486) revealed 246 specifically regulated (73 up- and 173 down-regulated) stromal GR-target genes (Fig. 1A, B). Next, we assessed the epithelial GR target gene signature after Dex treatment in androgen ablated and 2^nd^ generation anti-androgen insensitive abl-Abi and abl-Enza cells. In these two cell lines, 149 specifically regulated (120 up- and 29 down-regulated) epithelial GR target genes were identified (Fig. 1C, D). Interestingly, pathway analysis revealed a similar enrichment of the GR and AR signatures in all datasets upon Dex treatment (Fig. S1 A, B), which confirms the link between GR activity and the AR response signature in investigated epithelial and stromal cells. To strengthen our microarray results, we searched for publicly available data and included the LREX´ (LnCaP/AR Resistant to Enzalutamide Xenograft derived) dataset from Aurora et al [10]. These cells also exhibit high GR levels. Re-analysis including both epithelial datasets resulted in the identification of 27 mutual up-regulated GR downstream genes (Fig. 1E). Surprisingly, comparison of genes up-regulated in epithelial and stromal cells, revealed only four commonly regulated candidates (MAO-A, FKBP5, KLF9, ERRFI1), suggesting a distinct response to GC treatment in investigated epithelial and stromal cells (Fig. 1F), (Table S1).

### MAO-A is a directly up-regulated GR target gene

**Fig. 2:**
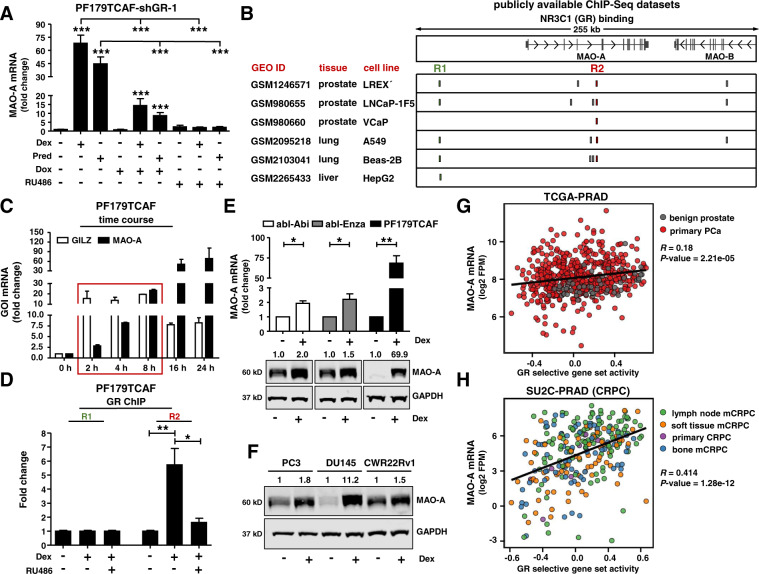
MAO-A is a directly regulated GR target and is associated with elevated GR activity. **A** MAO-A mRNA expression in PF179TCAF-shGR-1 cells after 24 h single or combination treatments with 100 nM Dex, 100 nM Pred, 1 µg/ml Dox, and 12 µM RU486 for 24 h. Data represent mean + SE from at least three independent experiments (one-way ANOVA and correction for multiple testing using Bonferroni´s comparison test; ***, *P* < 0.001). **B** Identification of 2 GR binding sites (R1 and R2) near the MAO-A gene in LREX´, LNCaP-1F5, VCaP, A549, Beas-2B, and HepG2 cells, screening publicly available ChIP-Seq datasets. **C** Time course of MAO-A and GILZ mRNA expression after incubation with 100 nM Dex. Data represent mean + SE from three independent experiments. **D** GR-ChIP with PF179TCAF cells after treatment with 100 nM Dex alone, or in combination with 6 µM RU486 for 16 h. Data represent mean + SE from three independent experiments (one-way ANOVA and correction for multiple testing using Bonferroni´s comparison test; *, *P* < 0.05). **E, F** MAO-A mRNA and protein expression after 100 nM Dex treatment for 3 d within different standard PCa cell models. mRNA data represent mean + SE from three independent experiments (unpaired *t*-test; *, *P* < 0.05; **, *P* < 0.01). **G, H** MAO-A is significantly positively correlated with GR activity within the publicly available TCGA-PRAD and SU2C-PRAD datasets.

MAO-A is a therapeutic target for the treatment of affective disorders and MAO-A inhibitors are routinely used for this indication in clinical practice [18]. Thus, we selected MAO-A as the prime candidate for follow-up experiments. In a first step, we confirmed our microarray results. MAO-A mRNA expression was significantly induced in PF179TCAF-shGR-1 cells within 24 h after GC treatment. This effect was abolished by simultaneous addition of shRNA mediated GR knockdown (Dox) or by pharmacological GR inhibition with RU486 (Fig. 2A). Next, we tested, if MAO-A is a primary target of the GR. Screening public available ChIP-Seq datasets for putative GR binding sites near or within the MAO-A gene revealed two predicted DNA binding regions (R1 and R2) in multiple cell lines of different origin (Fig. 2B). In addition, a time course experiment demonstrated early MAO-A induction even after 2 h Dex treatment (Fig. 2C). This regulation resembles GC regulation of GILZ, a well-known primary GR-target. To verify ChIP-Seq data predictions, a GR-ChIP was performed with PF179TCAF (Fig. 2D and Fig. S1 C). Dex treatment resulted in significantly elevated binding of the activated GR at region R2, which was blocked by RU486. Taken together, this demonstrates that MAO-A is a direct GR target gene.

### GC treatment results in elevated epithelial and stromal MAO-A protein expression

**Fig. 3:**
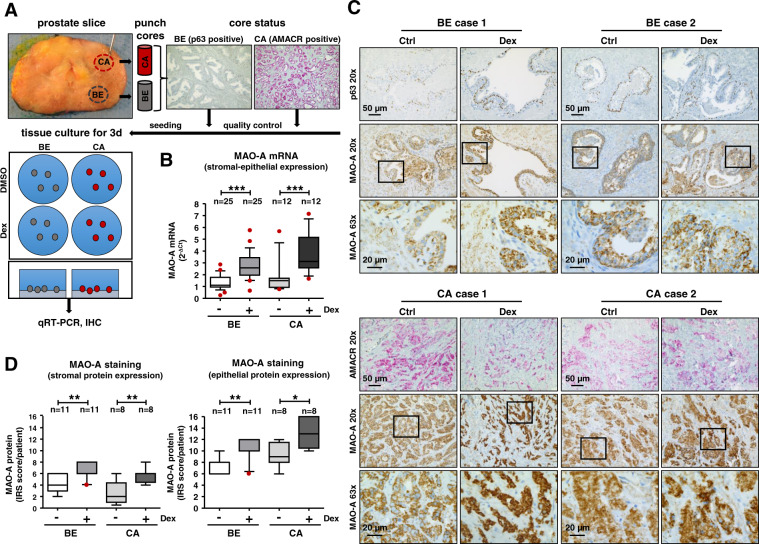
Evaluation of MAO-A expression in benign and cancerous tissue cores of RPE patients after GC treatment. **A** Schematic illustration of the ex vivo tissue culture. Punching, quality control by IHC staining for benign epithelium (p63) and for tumor tissue (AMACR) and cultivation of processed prostate tissue pieces in 6-well tissue culture plates. **B** MAO-A mRNA expression after 72 h incubation with 100 nM Dex. Data represent mean + SE from 25 benign and 12 cancerous screened tissue cores (one-way ANOVA and correction for multiple testing using Bonferroni´s comparison test; *, *P* < 0.05; ***, *P* < 0.001; Box Whisker Plot with 10–90 percentile). **C** Representative microscopy images of p63-AMACR and MAO-A staining of benign and cancerous tissue cores. Magnification: 20× (scale bar = 50 µm) and 63× (scale bar = 20 µm). **D** Quantification of MAO-A IRS after IHC staining of the epithelial and stromal compartment within benign and cancerous tissue cores (Kruskal–Wallis test and correction for multiple testing using Dunn´s comparison test; *, *P* < 0.05; **, *P* < 0.01; Box Whisker Plot with 10–90 percentile).

Due to GR mediated regulation of MAO-A on mRNA level, we aimed to elucidate if GC treatment also affects protein expression. The specificity of the MAO-A antibody was confirmed (Fig. S2 A-E). Notably, significantly elevated MAO-A mRNA and protein levels were observed in all cell lines used in the microarray screen (Fig. 2E) as well as in multiple PCa cell models (Fig. 2F) after 3 days GC treatment (Fig. S2 F). To avoid biased results induced by immortalization of used cell models, we additionally investigated MAO-A expression in PCa tissues. A screen of the publicly available TCGA-PRAD (mostly untreated primary PCa and adjacent benign samples) (Fig. 2G) and SU2C-PRAD [(heavily treated primary castration resistant prostate cancer (CRPC) and metastatic castration resistant prostate cancer (mCRPC) samples)] (Fig. 2H) datasets revealed that MAO-A mRNA expression is significantly positively correlated with GR activity. Of note, this correlation is stronger in CRPC samples. The importance of these findings was further verified using ex vivo tissue cultures of benign and cancerous primary tissue cores of PCa patients undergoing RPE (Fig. 3A). Dex treatment for 3 days resulted in significantly increased MAO-A mRNA expression in benign as well as in cancerous tissue samples (Fig. 3B). Notably, MAO-A IHC staining (Fig. 3C) and quantification (Fig. 3D) revealed not only significantly elevated MAO-A protein levels in the epithelial compartment, but also in the tumor stroma upon Dex treatment of the ex vivo cultures.

### MAO-A expression is induced during neoadjuvant chemotherapy

**Fig. 4:**
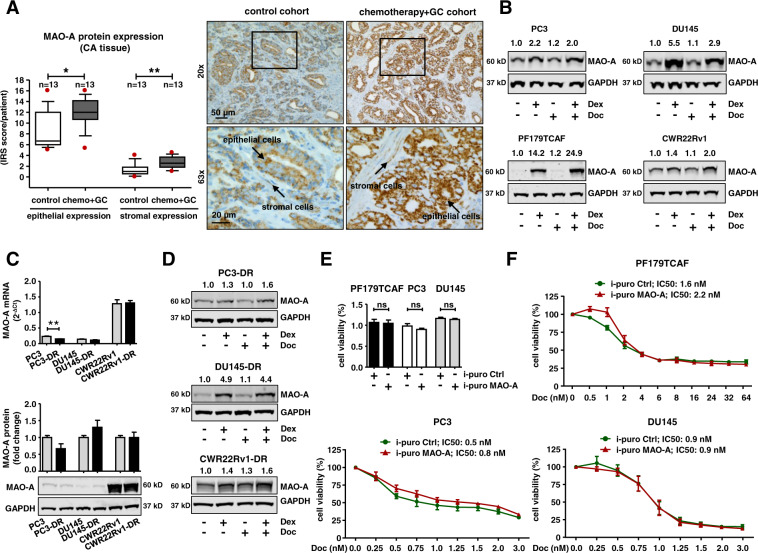
Screening of MAO-A expression after chemotherapeutic treatment. **A** Quantification of epithelial and stromal MAO-A IRS within cancerous areas of 13 selected chemotherapy and control patients (Mann–Whitney test; *, *P* < 0.05; **, *P* < 0.01; Box Whisker Plot with 10–90 percentile) and representative microscopy images. Magnification: 20× (scale bar = 50 µm) and 63× (scale bar = 20 µm). **B** Representative western blots for MAO-A protein expression after single treatment with either 100 nM Dex or 3 nM docetaxel, or combination of both drugs for 72 h within different cell models. **C** Basal MAO-A mRNA and protein expression in different parental and docetaxel resistant cell models. mRNA data represent mean + SE from three independent experiments (unpaired *t*-test; **, *P* < 0.01). **D** Representative western blots for MAO-A protein expression, after single treatment with either 100 nM Dex or 12.5 nM docetaxel, or combination of both drugs for 72 h within different docetaxel resistant cell models. **E** Basal cell viability after 5 days for PF179TCAF, PC3, and DU145 cells containing a constitutive MAO-A overexpression or control vector. **F** Cell viability screen after 5 days for PF179TCAF, PC3, and DU145 cells containing a constitutive MAO-A overexpression or control vector in the presence of increasing concentrations of docetaxel.

Next, we sought clinical confirmation for our observed in vitro and ex vivo tissue results. A previously described cohort with surgical specimens from 13 PCa patients who received neoadjuvant docetaxel chemotherapy combined with GC administration before RPE were screened for MAO-A expression, and compared to 13 treatment-naïve RPE control patients [19]. Of note, patient tissue analyses revealed a significantly elevated epithelial and stromal MAO-A expression in primary PCa tissue (Fig. 4A) as well as in corresponding benign tissue samples (Fig. S3 A) of chemotherapy patients compared to the control cohort.

As adjuvant GC treatment seems to have a major impact on GR signaling and in consequence also on MAO-A expression, we wanted to elucidate in a next step, if elevated epithelial and stromal MAO-A levels are a consequence of GC alone, or a combined effect of docetaxel and GCs. Unfortunately, comprehensive patient cohorts were not available to answer this question. Therefore, PC3, DU145, CWR22Rv1, and PF179TCAF cells were treated with either Dex or docetaxel alone, or with a combination of both drugs for 3 days (Fig. 4B). Docetaxel treatment had no significant impact on MAO-A protein, whereas Dex treatment resulted in elevated MAO-A levels. Interestingly, combination treatment resulted in an additive effect only in PF179TCAF, concluding that short time docetaxel treatment does not significantly affect MAO-A levels in the investigated epithelial cells. To evaluate possible chemotherapy long-term effects on MAO-A expression we also screened previously established long-term docetaxel (but not GC) treated and resistant PC3-DR, DU145-DR and CWR22Rv1-DR cells. Docetaxel insensitive cells did not exhibit significantly induced MAO-A basal expression on mRNA or protein level (Fig. 4C). GC treatment, however, resulted again in elevated MAO-A protein levels, suggesting that GCs are responsible for significantly induced MAO-A expression in epithelial cells during chemotherapy regimens (Fig. 4D).

### MAO-A overexpression does not lead to chemotherapy insensitivity, EMT, or induced stemness

As chemotherapy patients exhibit high MAO-A levels we wanted to further elucidate if MAO-A is able to promote chemotherapy insensitivity. To address this question, constitutive MAO-A overexpressing DU145, PC3, and PF179TCAF cell sub-lines were generated (Fig. S3 B). MAO-A overexpression did not result in a different cell phenotype (Fig. S3 C) nor in an altered cell viability (Fig. 4E). Furthermore, MAO-A overexpressing DU145, PC3, and PF179TCAF cell sub-lines were as sensitive as control cell lines when treated with increasing docetaxel concentrations (DU145 i-puro MAO-A/Ctrl, IC50: 0.9 nM/0.9 nM Doc; PC3 i-puro MAO-A/Ctrl, IC50: 0.5 nM/0.8 nM Doc; PF179TCAF i-puro MAO-A/Ctrl, IC50: 1.6 nM/2.2 nM Doc) (Fig. 4F). Previously, we correlated EMT and an emerging “stem cell like” CD24^low^-CD44^high^ cell sub-population with acquired chemotherapy resistance [19]. Recent publications reported that MAO-A overexpression might also play a significant role within this process [17, 20]. To investigate EMT after MAO-A overexpression, we performed a qRT-PCR screen for well-known epithelial/mesenchymal markers like E-cadherin, N-cadherin, EpCAM, Zeb1, and Vimentin (Fig. S4 A). Interestingly, aside from the observed cell line specific regulation of different investigated markers, MAO-A overexpression or MAO-A knockdown did not result in an EMT on mRNA or protein level (Fig. S4 B, C). Surprisingly, we instead observed a stabilization of the epithelial phenotype. Furthermore, a qRT-PCR screen for well-known stem cell markers like CD44, CD49b, Nanog, and CXCR4 did not indicate increased stemness in MAO-A overexpressing cells (Fig. S5 A). In concordance with qRT-PCR results, flow cytometry analysis revealed a reduction in CD44 expression in DU145 cells, as well as a diminished CD24^low^-CD44^high^ cell sub-population (Fig. S5 B-D). In conclusion, this demonstrates that MAO-A overexpression does not lead to an EMT or an induced stem cell like phenotype in our investigated cell models.

### MAO-A basal expression is significantly elevated during PCa progression and is correlated with reduced progression-free survival

**Fig. 5:**
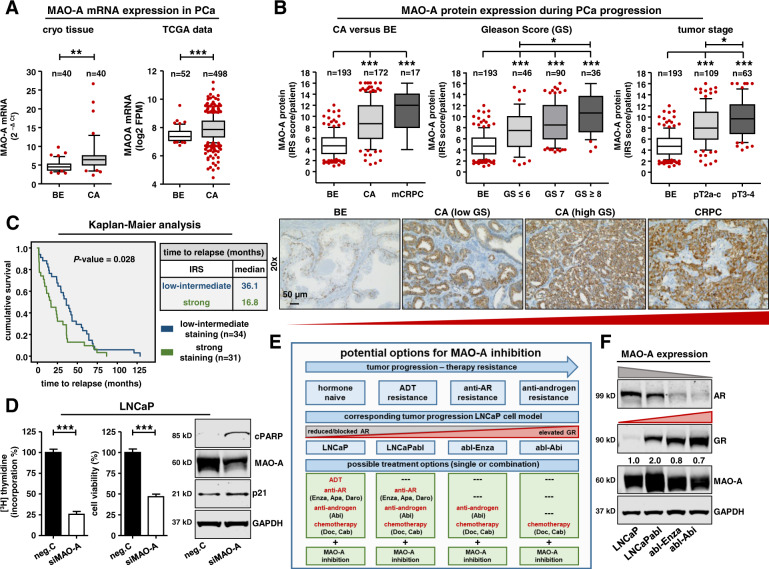
Significantly elevated MAO-A expression during tumor progression. **A** MAO-A mRNA expression in benign and cancerous cryo tissue samples of 40 primary PCa specimens and MAO-A mRNA expression of 52 benign and 498 cancerous samples from the TCGA database (unpaired *t*-test; **, *P* < 0.01; ***, *P* < 0.001, Box Whisker Plot with 10–90 percentile). **B** Quantification of MAO-A IRS of 193 benign, 172 primary and 17 CRPC resection tissue samples as well as further stratification in low GS (≤6), intermediate GS [7], high GS (≥8) patients and in different tumor stages (Kruskal–Wallis test and correction for multiple testing using Dunn´s comparison test; *, *P* < 0.05;***, *P* < 0.001, Box Whisker Plot with 10–90 percentile). Representative microscopy images. Magnification: 20× (scale bar = 50 µm). **C** Kaplan–Maier analysis of progression-free survival (progression is defined as re-rising PSA levels (>0.2 ng/ml) within at least two consecutive measurements) of tumor relapse patients with low/intermediate (IRS ≥ 10) or strong (IRS < 10) MAO-A expression in tumor sections [Log Rank (Mantel Cox); *P* value: 0.028]. **D** Measurement of cell proliferation and cell viability as well as western blot analysis for cPARP and p21 protein expression after transient transfection with either 25 nM siMAO-A or scrambled neg.C for 9 d. Data represent mean + SE from three independent experiments (unpaired *t*-test; ***, *P* < 0.001). **E** Schematic illustration of tumor progression. Current treatment options for different progression steps and the combination with potential pharmacological MAO-A inhibition in LNCaP and corresponding resistant LNCaP cell sub-lines as representative preclinical in vitro treatment models. **F** Representative western blot analysis for AR, GR and MAO-A protein expression in hormone naïve parental and therapy resistant LNCaP cells.

Next, we hypothesized that elevated MAO-A expression is associated with tumor aggressiveness and that targeting this protein might have a therapeutic effect. To answer this question, we identified the basal MAO-A expression pattern in benign prostate tissue specimens as well as in tumor samples representing different tumor stages during PCa progression. For a detailed overview, we screened publicly available single cell RNAseq data of normal and BPH prostate glands (Fig. S6 A). MAO-A mRNA expression was prominent in basal and luminal epithelial cells, and hillock (epithelial sub-cell type; KRT5^+^/KRT14^−^/KRT13^+^) cells. Conversely, club (epithelial sub-cell type; KRT5^−^/KRT8^−^/SCGB1A1^+^), neuro-endocrine, fibroblast, smooth muscle, endothelial, and leukocyte cells had weaker expression. Next, we used cryo-conserved macro-dissected tissue specimens from 40 hormone-naïve PCa patients and observed significantly elevated MAO-A mRNA expression in cancerous compared to benign samples (Fig. 5A). Elevated MAO-A mRNA expression was additionally confirmed with the TCGA database (prostate dataset, 52 benign versus 498 PCa samples) (Fig. 5A), as well as with the Oncomine datasets (Fig. S6 B, C). For PCa, the *Luo-Prostate* [21], *Singh-Prostate* [22], *Vanja-Prostate* [23], and *Welsh-Prostate* [24] datasets showed significantly elevated MAO-A gene expression in cancerous tissues. Furthermore, observations at mRNA level were successfully translated to the protein level. MAO-A IHC analysis in benign prostate glands revealed intense MAO-A staining in basal epithelial cells followed by a low to intermediate staining in luminal epithelial and stromal cells (Fig. S7 A). In cancer tissue, stromal MAO-A staining remained unchanged, while it was elevated in luminal cells. Statistical analysis of PCa patients (table S2) revealed a significantly induced MAO-A protein expression in primary cancer and mCRPC resection tissue compared to benign samples (Fig. 5B). Subsequent stratification in low Gleason score (GS) (≤6), intermediate GS [7] and high GS (≥8), as well as in low and high tumor stage cases, revealed MAO-A levels significantly increased with PCa aggressiveness (Fig. 5B). In addition, we assessed the association of MAO-A expression in tumor tissue with time-to-biochemical relapse. In a cohort of 65 patients (table S3) with confirmed biochemical relapse within 11 years after RPE, those with high (IRS > 10) MAO-A expression had a significantly shorter time-to-relapse (median 16.8 mo) than patients with low-intermediate (IRS ≤ 10) MAO-A expression (median 36.1 mo) (Fig. 5C), confirming the importance of elevated MAO-A expression for accelerated tumor progression.

With regard to cellular models to test the therapeutic effect of MAO-A targeting, we performed an in vitro cell line screen for MAO-A mRNA and protein expression (Fig. S7 B, C). Benign epithelial and stromal cell lines supported the MAO-A expression pattern within benign prostate glands. However, epithelial cancer cell lines showed a heterogeneous expression pattern. AR positive cell lines like LAPC4, VCaP, DUCaP, CWR22Rv1, LNCaP, and LNCaPabl exhibited intermediate to intense MAO-A expression, whereas AR negative PC3 and DU145 cells had a low to intermediate expression. This expression pattern indicates regulation of MAO-A also by the AR. Analysis of the TCGA-PRAD and SU2C-PRAD (Fig. S8 A) datasets indeed revealed that MAO-A mRNA expression is significantly positively correlated with AR activity, similar to observed GR activity in primary and metastatic cancer (Fig. 2G, H). In addition, we have used these datasets for GSEA pathway analysis. Notably, MAO-A was significantly correlated with the MSigDB hallmark androgen response pathway (Fig. S8 B). Furthermore, screening of publicly available AR and GR ChIP-Seq datasets for LREX´, LNCaP-1F5, and VCaP cells identified identical AR and GR binding sites in the MAO-A gene, confirming the clinical importance of the shared GR-AR signaling axis for PCa (Fig. S8 C).

### MAO-A targeting is effective in hormone naïve, hormone ablated, and long-term anti-androgen treated PCa cells

**Fig. 6:**
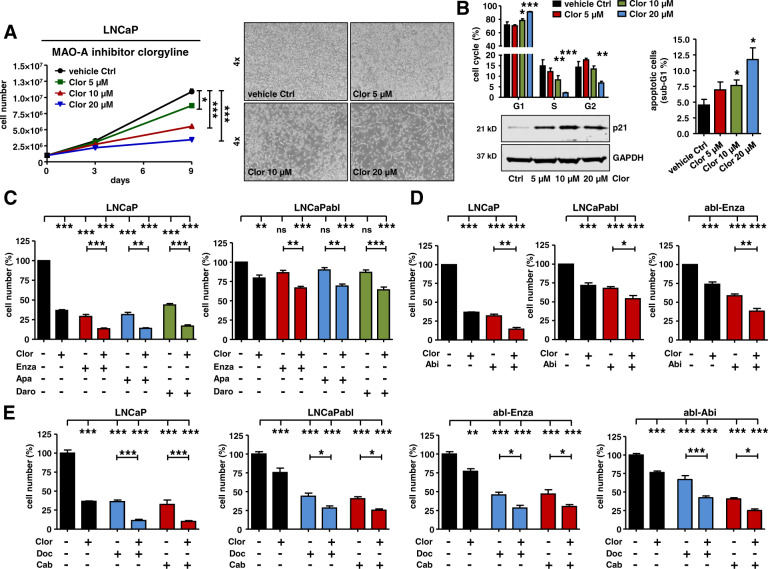
Consequences of MAO-A inhibition for PCa cell growth and viability. **A** Measurement of LNCaP cell growth and representing cell pictures after 9 d treatment with increasing concentrations (5 µM, 10 µM, 20 µM) of the specific MAO-A inhibitor clorgyline. Data represent mean + SE from four independent experiments (one-way ANOVA and correction for multiple testing using Dunnett´s comparison test; *, *P* < 0.05; ***, *P* < 0.001). **B** Measurement of cell cycle distribution and percentage of apoptotic cells, as well as western blot analysis for p21 protein expression after treatment with increasing clorgyline concentrations for 9 d. Data represent mean + SE from four independent experiments (one-way ANOVA and correction for multiple testing using Dunnett´s comparison test; *, *P* < 0.05; **, *P* < 0.01; ***, *P* < 0.001). **C** LNCa*P* and LNCaPabl cell growth measurement after 9 d treatment with 10 µM clorgyline alone or in combination with 2.5 µM enzalutamide, apalutamide, or darolutamide. Data represent mean + SE from at least three independent experiments (one-way ANOVA and correction for multiple testing using Bonferroni´s comparison test; **, *P* < 0.01; ***, *P* < 0.001). **D** LNCaP, LNCaPabl, and abl-Enza cell growth measurement after 9 d treatment with 10 µM clorgyline alone or in combination with 2.5 µM abiraterone. Data represent mean + SE from at least three independent experiments (one-way ANOVA and correction for multiple testing using Bonferroni´s comparison test; *, *P* < 0.05; **, *P* < 0.01; ***, *P* < 0.001). **E** LNCaP, LNCaPabl, abl-Enza, abl-Abi cell growth measurement after 9 d treatment with 10 µM clorgyline alone or in combination with 0.4 nM (for LNCaP, LNCaPabl, abl-Enza) or 0.75 nM (for abl-Abi) docetaxel or cabazitaxel. Data represent mean + SE from at least three independent experiments (one-way ANOVA and correction for multiple testing using Bonferroni´s comparison test; *, *P* < 0.05; **, *P* < 0.01; ***, *P* < 0.001).

As MAO-A expression is elevated in PCa tissue during all tumor stages we explored the effect of MAO-A targeting. As expected, transient MAO-A knockdown in hormone naïve LNCaP cells resulted in significantly reduced cell proliferation and cell viability as well as induction of the cell cycle inhibitor p21 and the apoptosis marker cPARP (Fig. 5D). Reduced cell viability and cell proliferation was additionally confirmed in corresponding hormone ablated LNCaPabl, and in enzalutamide and abiraterone long-term treated abl-Enza and abl-Abi cells (Fig. S8 D). These cell-subline models represent different clinical resistance stages during tumor progression and are characterized by blocked or decreased AR and elevated GR levels (Fig. 5E, F). Observed effects of MAO-A knockdown on cell viability and cell proliferation are in concordance with already published in vitro and in vivo data using different PCa cell lines [17, 25–27]. To further evaluate the potential use of MAO-A targeting for a clinical approach, pharmacological MAO-A inhibition was performed. Consecutive treatment with the specific MAO-A inhibitor clorgyline for 9 days resulted in a dose dependent reduction in cell growth in all tested cell models, with the strongest effect seen in hormone naïve LNCaP cells (Fig. 6A and Fig. S9 A). Flow cytometry analysis after clorgyline treatment revealed a cell cycle G1 arrest, which was confirmed by increased p21 expression and elevated apoptosis (Fig. 6B). Furthermore, clorgyline treatment was combined with 2^nd^ generation AR signaling inhibitors. As expected, LNCaP cells were sensitive to all anti-androgens when compared to ADT resistant LNCaPabl. Combination treatment with clorgyline was superior to single anti-androgen treatment and resulted in significantly reduced cell growth (Fig. 6C). Combination treatment of abiraterone and clorgyline exhibited similar improvement in results, compared to single treatments in tested LNCaP, LNCaPabl and abl-Enza cells (Fig. 6D). Most notably, however, combined treatment of selected hormone naïve and therapy resistant cell models with clorgyline and docetaxel or cabazitaxel (Fig. 6E and Fig. S9 B), resulted in significantly reduced cell growth compared to chemotherapy alone.

## Discussion

The implementation of 2^nd^ generation AR signaling inhibitors or taxane based chemotherapeutics in addition to ADT monotherapy significantly prolonged PCa patient overall survival. However, an elemental part of several of these therapy regimens is additional GC treatment. GCs are routinely given to patients in combination with docetaxel, cabazitaxel [5, 12, 28], and abiraterone [13, 29] as they exert anti-inflammatory as well as anti-emetic effects. Their independent impact on survival, however, is unclear, and unfavorable effects such as osteoporosis and immunosuppression complicate long-term use [30]. Furthermore, the impact of GC treatment on genes of the stromal GR signaling pathway, which may additionally influence tumor progression and therapy resistance, is unknown.

In order to close this gap of knowledge, we have elucidated the altered epithelial and stromal GR signature after GC treatment and identified MAO-A as a significantly up-regulated mutual epithelial and stromal GR target. An important paralog of this gene is MAO-B. Although, both proteins share a 70% amino acid sequence identity, our microarray screens indicate that MAO-B is not significantly affected by GC treatment, unlike MAO-A. Diseases already associated with MAO-A alterations include Brunner Syndrome [31] and an array of other behavioral and neurological disorders [32, 33]. Emerging evidence has also implicated MAO-A expression in the development of PCa related chemotherapy resistance [16]. Gordon et al. reported induced MAO-A levels after chemotherapy with docetaxel or mitoxantrone. They concluded that MAO-A overexpression was a direct effect of the chemotherapeutics, and subsequently associated high MAO-A levels with chemotherapy resistance. Based on our data, we were able to confirm and extend upon these findings. We observed not only significantly elevated MAO-A levels in benign and malignant tissue samples of neoadjuvant docetaxel treated PCa patients but also in CAFs within the tumor stroma. Furthermore, additional in vitro screens with multiple parental and docetaxel resistant cells revealed that the adjuvant GC medication used with chemotherapy regimens causes significantly elevated MAO-A expression and not the chemotherapeutics themselves. Moreover, MAO-A overexpression did not result in heightened therapy insensitivity in investigated epithelial cell models. Surprisingly, we observed elevated MAO-A levels after combined GC and docetaxel treatment in investigated CAFs. These cells had a lower sensitivity to docetaxel compared to epithelial cells, suggesting that CAFs and used GCs might have an important role in acquired therapy resistance and tumor progression. Observed functional differences between epithelial and stromal cells might be explained by the fact that GC treatment elicited distinct specific activated GR gene signatures as seen in our microarray screens. In concordance with this hypothesis are other investigations reporting MAO-A dependent induced PCa metastases due to elevated tumor stroma cell interactions [27, 34].

Previously, we associated EMT and emerging stemness with acquired chemotherapy resistance [19]. Recent publications reported that MAO-A overexpression plays a significant role in both processes during tumor progression [17, 20, 34]. However, despite screening numerous well-known EMT and stem cell markers on mRNA and protein level, we were not able to observe EMT or elevated stemness after MAO-A overexpression. Rather, we observed a general stabilization of the epithelial phenotype and an unchanged or reduced CD44 overall expression as well as an unchanged or reduced CD24^low^ and CD44^high^ cell-sub population in investigated PC3 and DU145 cells. Our in vitro findings are further supported by a GSEA pathway analysis of publicly available patient datasets. Screening of the TCGA-PRAD and SU2C-PRAD datasets revealed a significantly negative correlation between MAO-A expression and the EMT pathway in primary and metastatic cancer. Surprisingly, MAO-A mRNA expression was also negatively correlated with the hypoxia and IL6-JAK-STAT3 pathways in both datasets previously associated with elevated MAO-A expression [17, 27]. Interestingly however, MAO-A was significantly positively correlated with oxidative phosphorylation, adipogenesis, and fatty acid metabolism - key-pathways connected with mitochondrial activity. It is known that metabolic alterations and a high load of non-synonymous mtDNA mutations are accompanied by an increase in mtDNA content and mt-density, which is a characteristic of aggressive PCa [35, 36]. In concordance, previously published findings identified MAO-A as a key gene within a set of 11 highly correlated metagenes extracted from the gene expression profile of PCa samples exhibiting a severe respiratory phenotype. Notably, these gene-sets were predictive of significantly shorter survival [37]. Based on these observations, it comes as little surprise that induced MAO-A expression was not only observed during tumor progression but also recently in circulating tumor cells (CTCs) [17, 38]. In line with these findings, we observed significantly elevated MAO-A expression in primary cancer and mCRPC resection material, and associated high MAO-A levels with a significantly earlier biochemical recurrence. Notably, we also correlated MAO-A expression with significantly elevated GR and AR activity, and the androgen response pathway, in primary and metastatic PCa samples. It has been consistently shown that both receptors not only exhibit a significant overlap in their transcriptome [10] but also share several interacting proteins [39]. Recently, Wang et al. reported that the AR splice variant ARv7 can bind to an ARE element within the MAO-A promotor region, with even higher affinity compared to full length AR [25]. ARv7 is the most common AR variant and it is associated with CRPC. Since no effective ARv7 targeting is available at the moment, pharmacological MAO-A inhibition might be a promising new tool to inhibit the combined GR-AR signaling axis. MAO inhibitors are already in use as standard treatment options for other medical indications [18]. Concerning their role in cancer treatment, MAO-A inhibitor monotherapy, or in combination with enzalutamide or docetaxel treatment, results in reduced proliferation and tumor growth in vitro and in vivo [16, 25, 40]. The MAO-A inhibitor phenelzine is used within an ongoing phase II clinical trial (NCT02217709) in patients with non-metastatic recurrent PCa. In concordance, we demonstrated a beneficial effect of MAO-A targeting for chemotherapy and ADT treatment. MAO-A inhibitor treatment, in combination with chemotherapeutics and 2^nd^ generation AR signaling inhibitors, resulted in significantly reduced cell proliferation and elevated apoptosis not only in hormone naïve and androgen ablated but more importantly in long-term abiraterone, and enzalutamide treated cells, supporting the importance of MAO-A targeting in all stages of advanced disease.

## Conclusion

In summary, the presented data identifies MAO-A as a promising mutual epithelial and stromal directly up-regulated GR target, which is significantly elevated in multiple PCa cell models and in primary PCa ex vivo tissue cultures after GC treatment, as well as in RPE specimens after neoadjuvant chemotherapy in combination with GCs. Furthermore, MAO-A expression is highly up-regulated during PCa progression. Pharmacological MAO-A inhibition in combination with anti-androgen or chemotherapy targets the GR-AR signaling axis and will therefore amplify standard-of-care medication for hormone naïve and mCRPC.

## Materials and methods

### Cell culture and chemicals

PC3, DU145, LNCaP, CWR22Rv1, and VCaP cells were obtained from the American Type Culture Collection (ATCC, Rockville, MD, USA). LAPC4 were a gift from Dr. A. Cato (University of Karlsruhe, Karlsruhe, Germany). BPH-1 and DUCaP cells were a gift from Dr. J. Schalken (Radboud University, Nijmegen, Netherlands). CWR22Rv1-DR cells were a gift from Dr. William Watson (University College Dublin, Dublin, Ireland). Cancer-associated fibroblasts (PF179TCAF), normal-associated fibroblasts (PF179TNAF), PM151T, RWPE-1, EP156T, BPH-1, PC3, DU145, CWR22Rv1, VCaP, DUCaP, LAPC4, PC3-DR, DU145-DR, LNCaP, LNCaPabl, LNCaPabl-Abi (abl-abi), and LNCaPabl-Enza (abl-Enza) were cultured as previously described [41]. The authenticity of all cell lines was validated via short tandem repeat (STR) profiling.

### Patient material and immunohistochemistry (IHC)

Patients were selected from the Innsbruck PCa biobank. The use of archived material was approved by the Ethics Committee of the Medical University of Innsbruck (Study no. AM 3174 including amendment 2). Written consent was obtained from all patients and documented in the database of the University Hospital Innsbruck in agreement with statutory provisions. Tissue microarrays (TMAs) containing benign and primary cancer tissue cores from 202 PCa patients who underwent open retropubic or robotic assisted (Da Vinci) RPE as well as resection material from mCRPC were employed to evaluate MAO-A expression. Matched benign samples were excised from histologically confirmed non-malignant regions of RPE specimens. In addition, 14 PCa patients who underwent neoadjuvant chemotherapy with docetaxel in combination with GC treatment before RPE, as well as 14 untreated PCa patients were selected from the Innsbruck PCa biobank and included within the MAO-A expression analysis. These patients have not received other chemotherapeutics or anti-androgens prior to RPE, and both groups were matched for GS and age [19]. MAO-A IHC was performed on a Ventana BenchMark device (Roche, Vienna, Austria). The following antibody was used: anti-MAO-A rabbit Mab (EPR7101, final dilution 1:1600) (Abcam, Cambridge, UK). Specificity of the used MAO-A antibody for IHC was confirmed by staining of PF179TCAF cells after treatment with either 100 nM Dex or with a combination of 100 nM Dex and 6 µM RU486 for 3 days. Cells were harvested and prepared for IHC staining as previously described [41]. TMAs were evaluated using the following modified “quick - score” protocol: staining intensity was scored 0–4 (0 = absent, 1 = weak, 2 = intermediate, 3 = strong, very strong = 4). The percentage of positively stained cells was scored 0–4 (0 = absent, 1 = <10%, 2 = <50%, 3 = <75%, 4 = >75%). Both scores were multiplied to obtain an immunoreactivity score (IRS), ranging from 0 to 16. For survival analysis, samples from patients with confirmed biochemical relapse within 11 years from RPE (*n* = 65; biochemical relapse = rising PSA levels (>0.2 ng/ml) in at least two subsequent measurements) at a known time point have been included. For that, patients have been divided into low-intermediate MAO-A (IRS ≥ 10) or high MAO-A (IRS < 10) groups, based on the MAO-A IRS in malignant tissue specimens.

### cDNA synthesis and qRT-PCR

cDNA synthesis was done with a Luna Script RT Super Mix Kit (New England Biolabs, Ipswich, USA) and qRT-PCR was performed on an ABI PRISM 7500-FAST system (ThermoFisher Scientific) using a Luna Universal Probe qPCR Master Mix (New England Biolabs) according to the manufacturer’s protocols. All expression data were normalized either to TBP (cell lines) or to the mean of TBP and HPRT1 (tissue samples). Custom primers were used at a concentration of 800 nmol/L each and FAM-TAMRA labeled probes at 150 nmol/L. Primer sequences for TBP and HPRT1 were previously described [42]. TaqMan gene-expression assays (ThermoFisher Scientific) were used for MAO-A (Hs00165140_m1), GILZ (Hs00608272_m1), E-cadherin (Hs01013955_m1), N-cadherin (Hs00983056_m1), EpCAM (Hs00901885_m1), Zeb1 (Hs00232783_m1), Vimentin (Hs00185584_m1), CD44 (Hs01075861_m1), CD49b (Hs00158127_m1), Nanog (Hs04399610_g1), and CXCR4 (Hs00607978_s1).

### Western blot

Cells were lysed in LDS sample buffer, 50 μg total protein was separated on NuPAGE Novex 4–12% Bis-Tris Protein Gels (ThermoFisher Scientific) and transferred to 0.2 μm nitrocellulose membranes (GE Healthcare, Vienna, Austria). The following antibodies were used: anti-MAO-A rabbit Mab (EPR7101, 1:2000) (Abcam), anti-E-cadherin (Cat:610181, 1:500) (Becton Dickinson, Heidelberg, Germany), Vimentin (SC6260, 1:500) (Cruz Biotechnology, Santa Cruz, CA), anti-GAPDH (MAB374, 1:50.000) (Millipore, Vienna, Austria), anti cPARP (G7341, 1:500) (Sigma), and anti-p21 (2946 S, 1:500) (Cell Signaling, Frankfurt, Germany). Specificity of the used MAO-A antibody was tested and confirmed using protein of PF179TCAF cells after treatment with either 100 nM Dex, or a combination of 100 nM Dex and 6 µM RU486 for 3 days and using protein of PC3 cells overexpressing MAO-A. Furthermore, reduced MAO-A protein was confirmed after transient MAO-A knockdown for 3 days in LNCaPabl cells utilizing ON-TARGET plus Human MAO-A siRNA SMARTpool (L-009369-00-0005), siControl On-Target plus Non-targeting pool (D-001810-10-05) and Lipofectamine2000 (ThermoFisher Scientific) according to the manufacturer’s protocols.

### Statistical analysis

SPSS (V24.0) and GraphPad Prism 8 were used for statistical analyses. For all experiments, Gaussian distribution was determined using Kolmogorov-Smirnov and D´Agostino & Pearson omnibus normality test. Differences between treatment groups were analyzed using unpaired Student’s *t* test or Mann–Whitney *U* test depending on Gaussian distribution. Comparison of multiple treatment groups was done using one-way Anova and corrected for multiple testing using Bonferroni or Dunn´s multiple comparison test method depending on Gaussian distribution. Correlation analysis was performed by the Spearman’rho method. Differences in recurrence-free survival were assessed using Kaplan–Meier plots and log-rank test. *P* values below 0.05 were considered significant. All differences highlighted by asterisks were statistically significant as encoded in figure legends (*P < 0.05, **P < 0.01, ***P < 0.001). Data are presented as mean + SE unless otherwise specified.

## Supplementary information

Figure S1

Figure S2

Figure S3

Figure S4

Figure S5

Figure S6

Figure S7

Figure S8

Figure S9

Additional File 1

Aditional File 2 Table S1

Additional File 3 Table S2, S3

Supplementary information

## Data Availability

All data generated or analyzed during this study are included in this published article and its supplementary information files are available in the NBCI Gene Expression Omnibus (GEO) repository, https://www.ncbi.nlm.nih.gov/gds.
