# Supplementary figures and images for: Targeting the glucocorticoid receptor signature gene Mono Amine Oxidase-A enhances the efficacy of chemo- and anti-androgen therapy in advanced prostate cancer
Source: Oncogene. 2021 Apr 1;40(17):3087–100. doi: 10.1038/s41388-021-01754-0 (PMC8084733; doi:10.1038/s41388-021-01754-0)

Figure S1

Gene enrichment signatures for GR and AR signaling

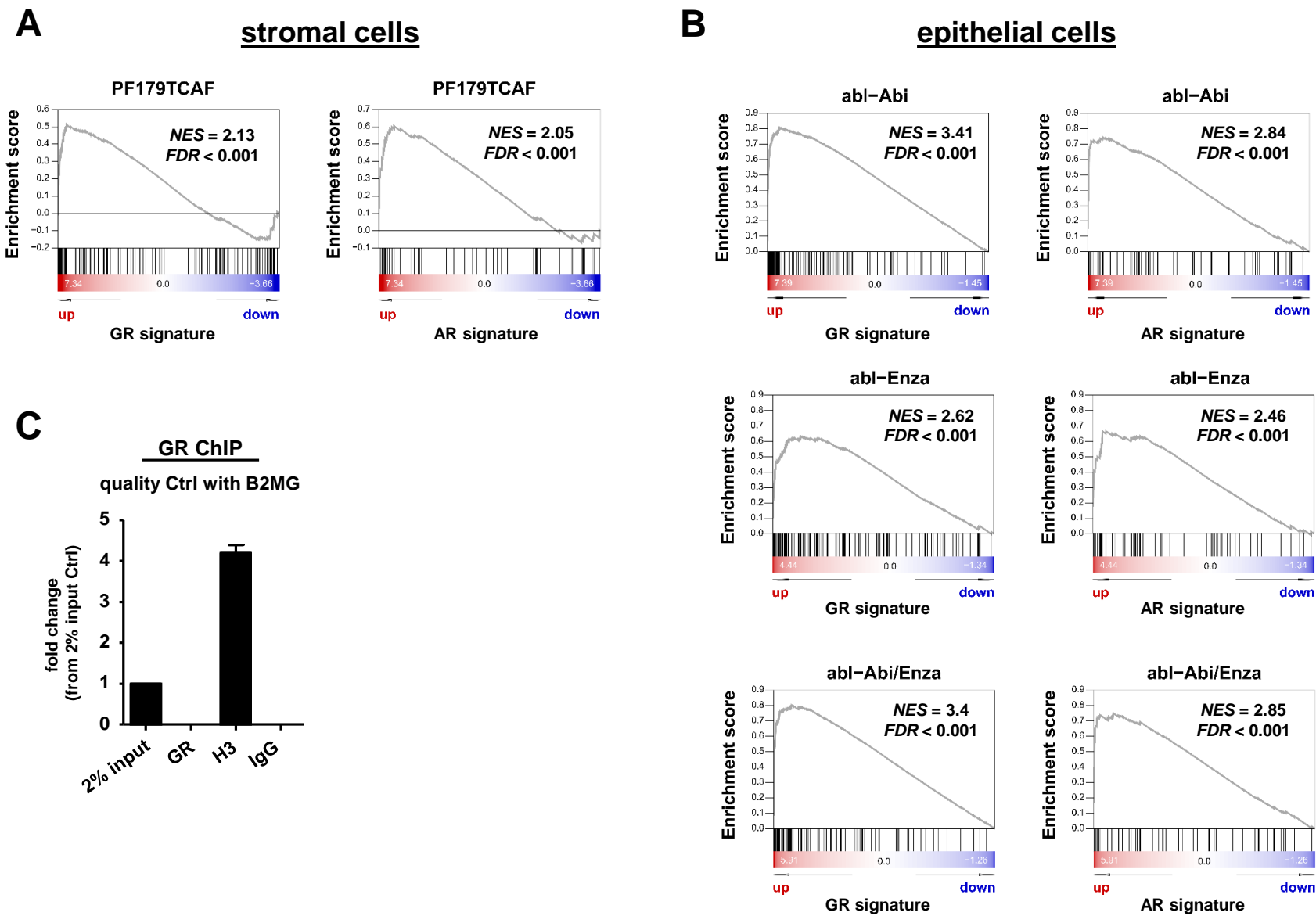

Supplement: Supplementary file 1 — Figure S1 [file 41388_2021_1754_MOESM1_ESM.pdf]

Figure S2

MAO-A antibody specificity testing for Western blot and IHC

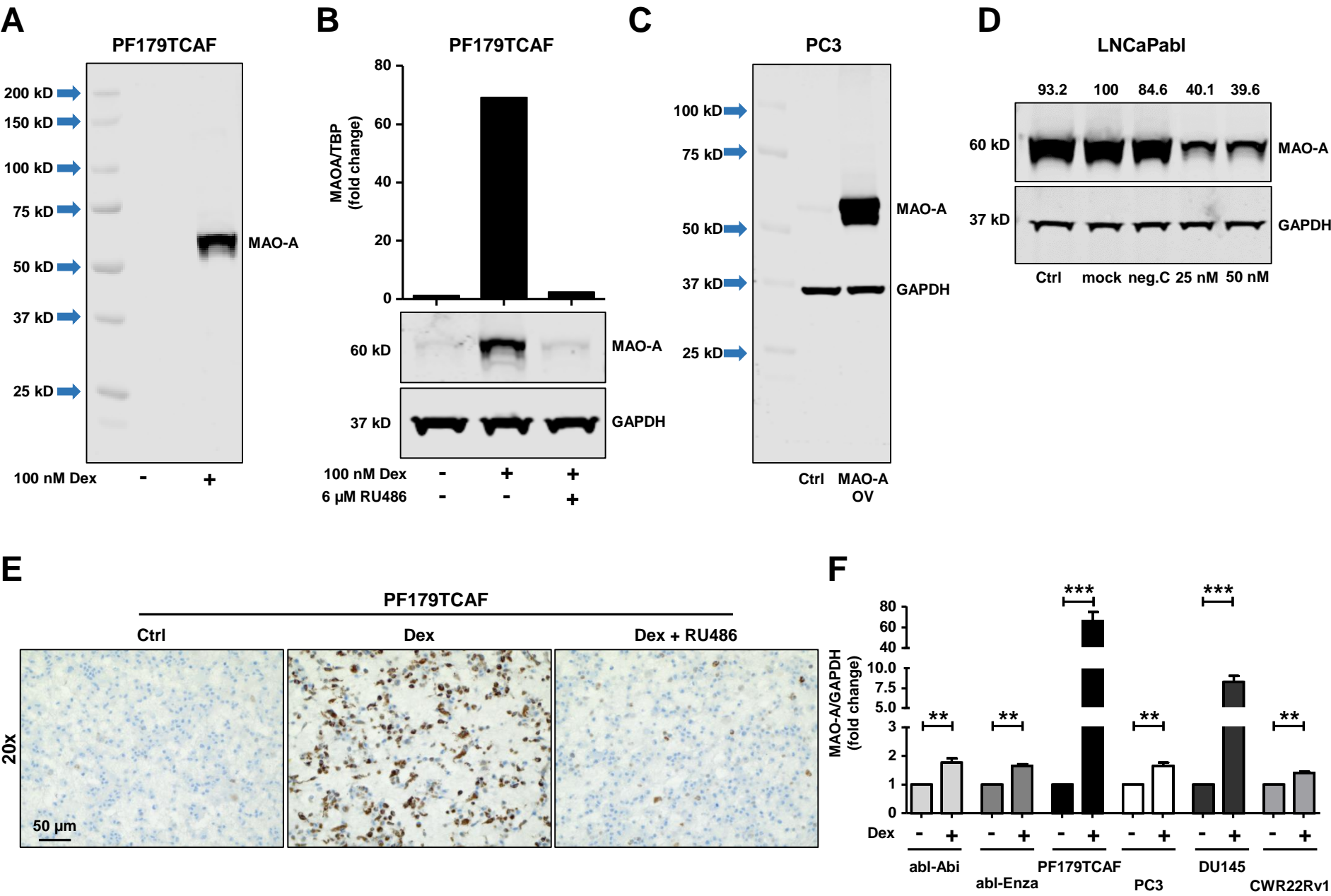

Supplement: Supplementary file 2 — Figure S2 [file 41388_2021_1754_MOESM2_ESM.pdf]

Figure S3

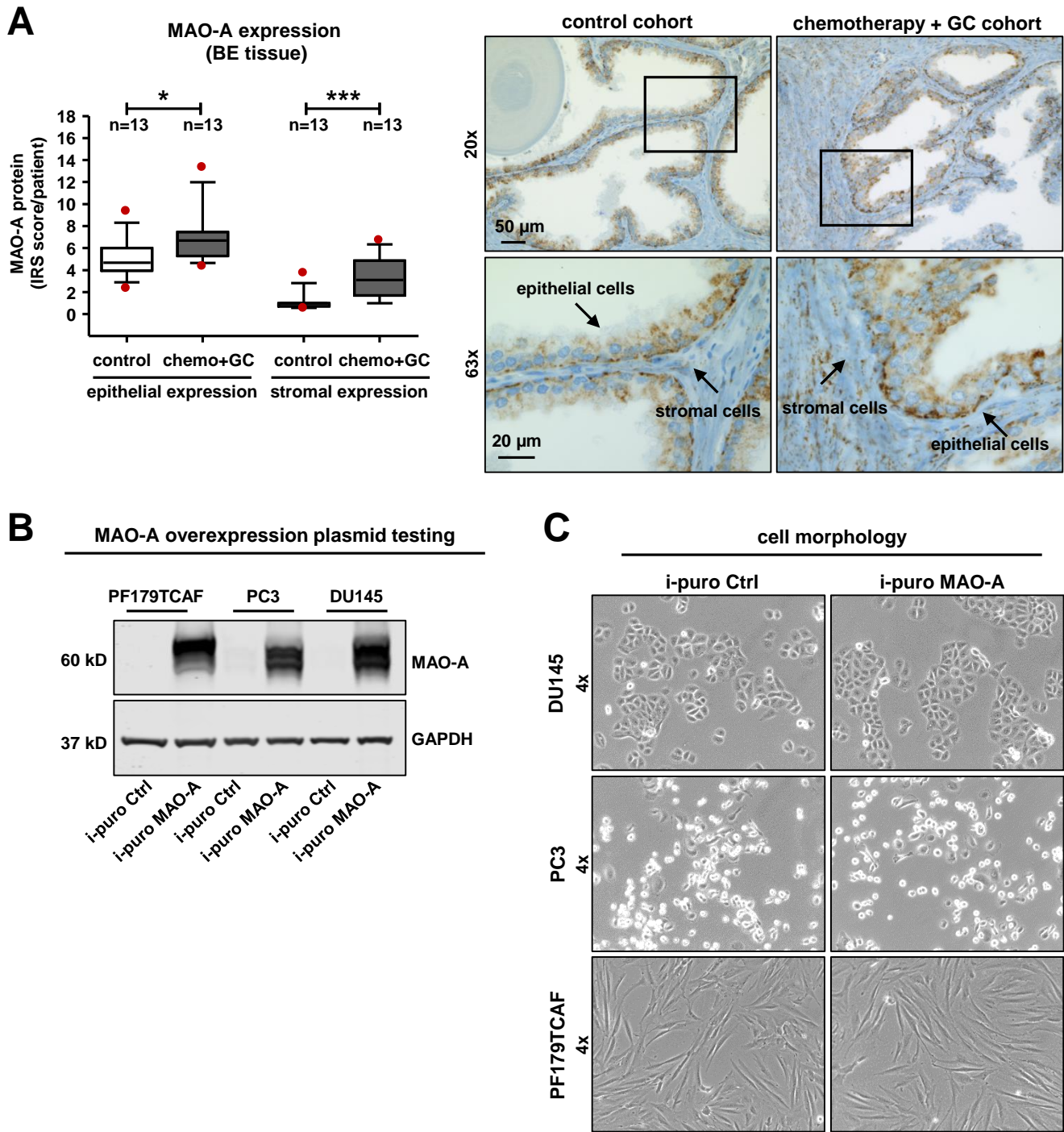

Supplement: Supplementary file 3 — Figure S3 [file 41388_2021_1754_MOESM3_ESM.pdf]

Figure S4

A

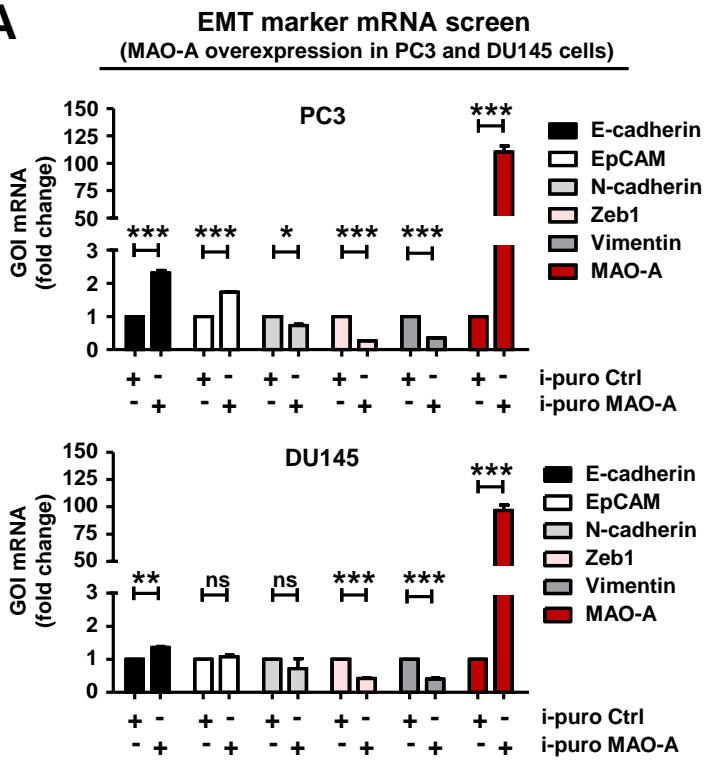

B

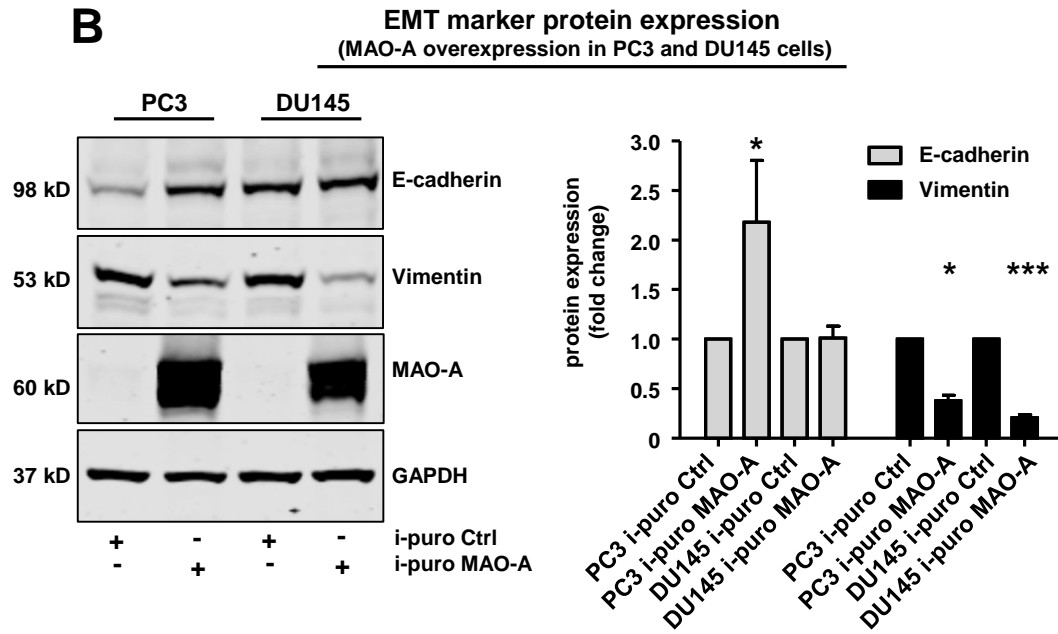

C

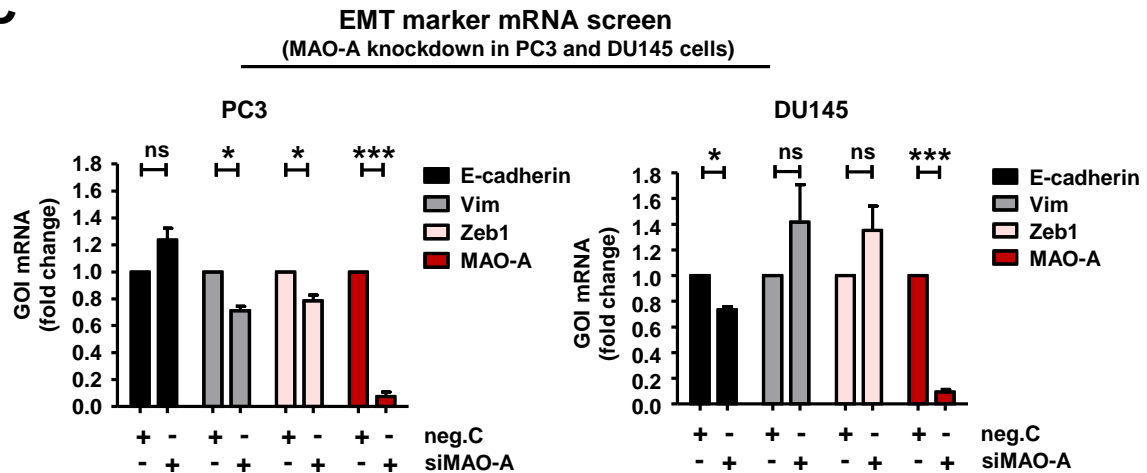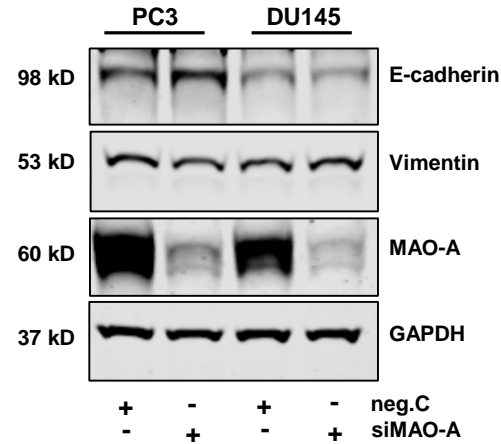

Supplement: Supplementary file 4 — Figure S4 [file 41388_2021_1754_MOESM4_ESM.pdf]

## Figure S5

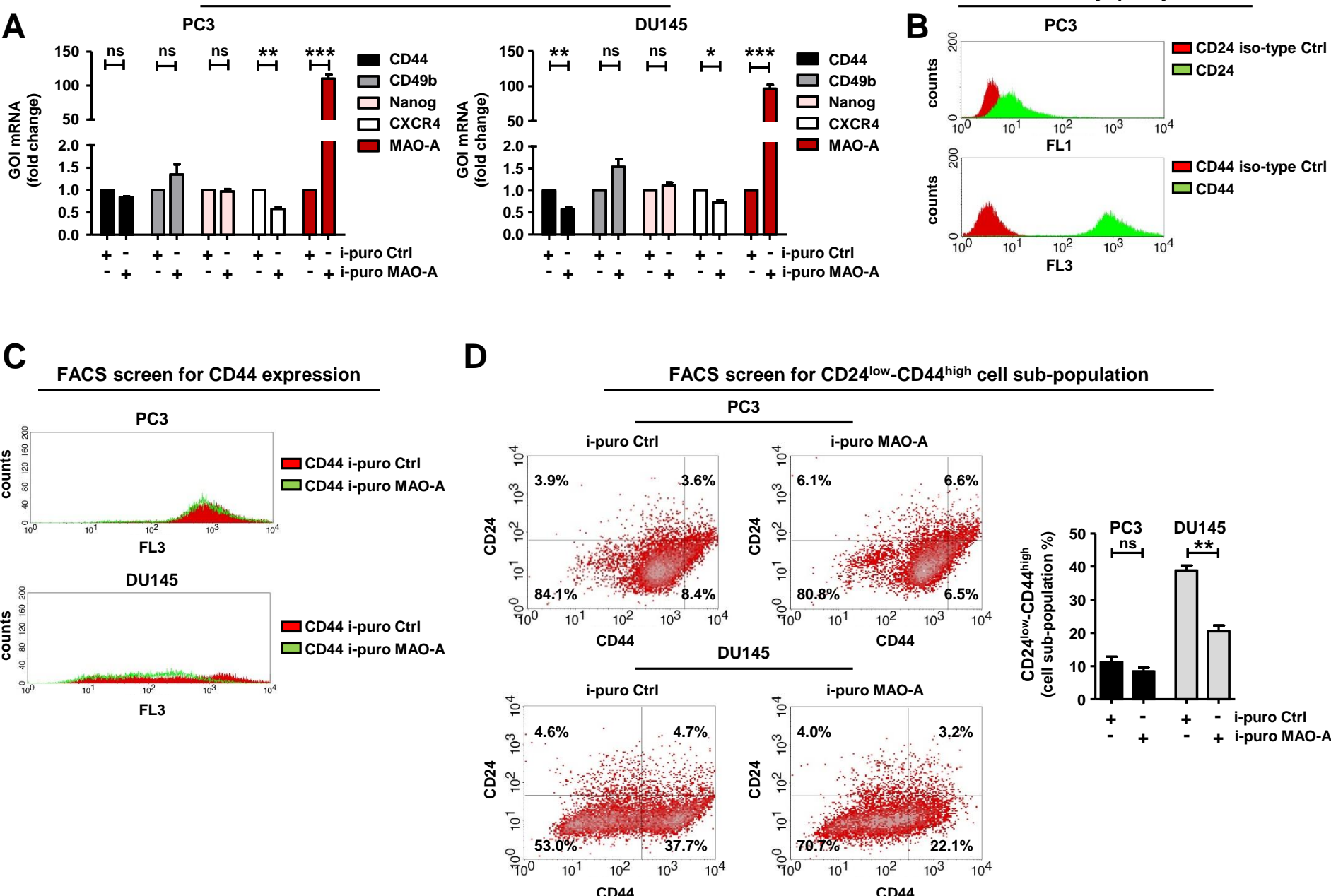

Supplement: Supplementary file 5 — Figure S5 [file 41388_2021_1754_MOESM5_ESM.pdf]

Figure S8

GSEA pathway analysis

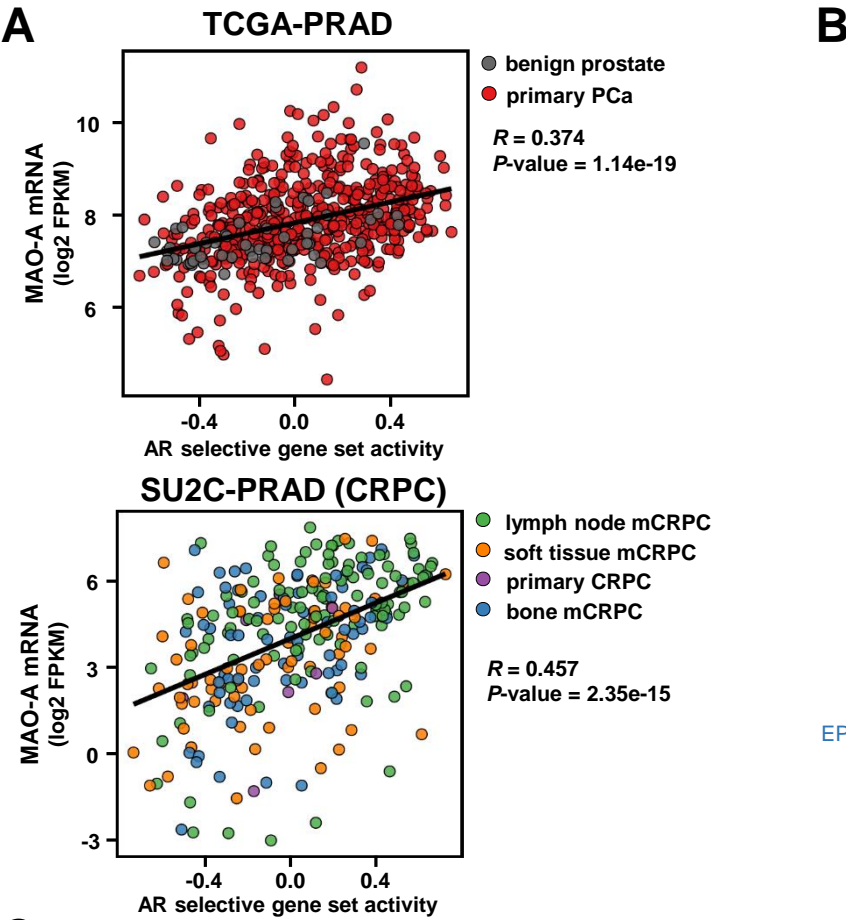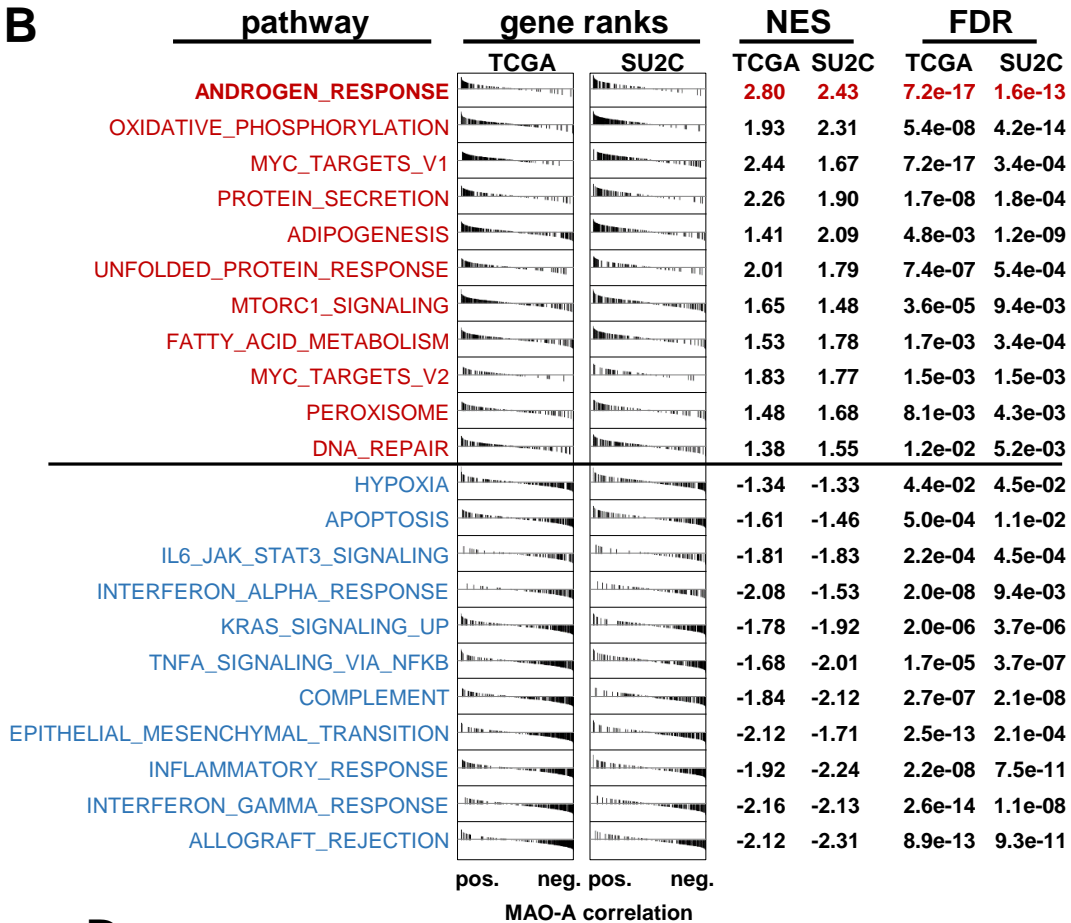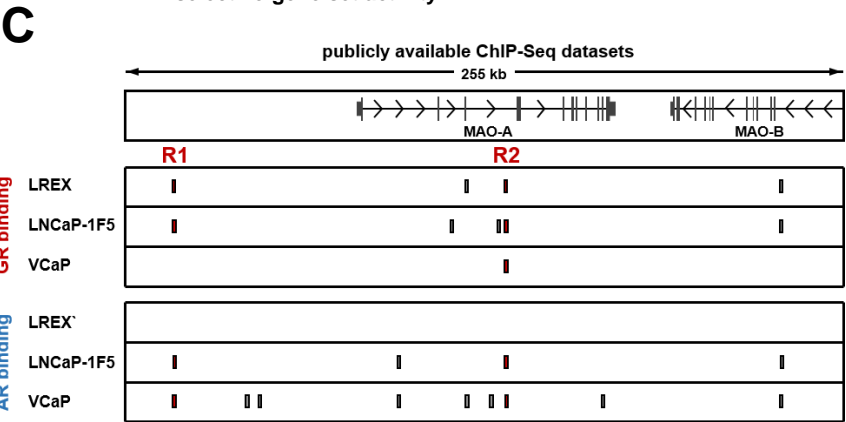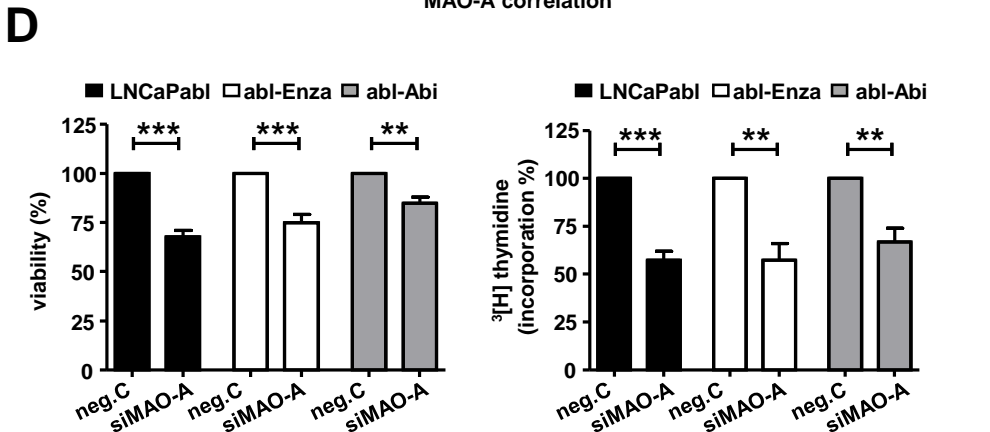

Supplement: Supplementary file 8 — Figure S8 [file 41388_2021_1754_MOESM8_ESM.pdf]

Figure S9

A

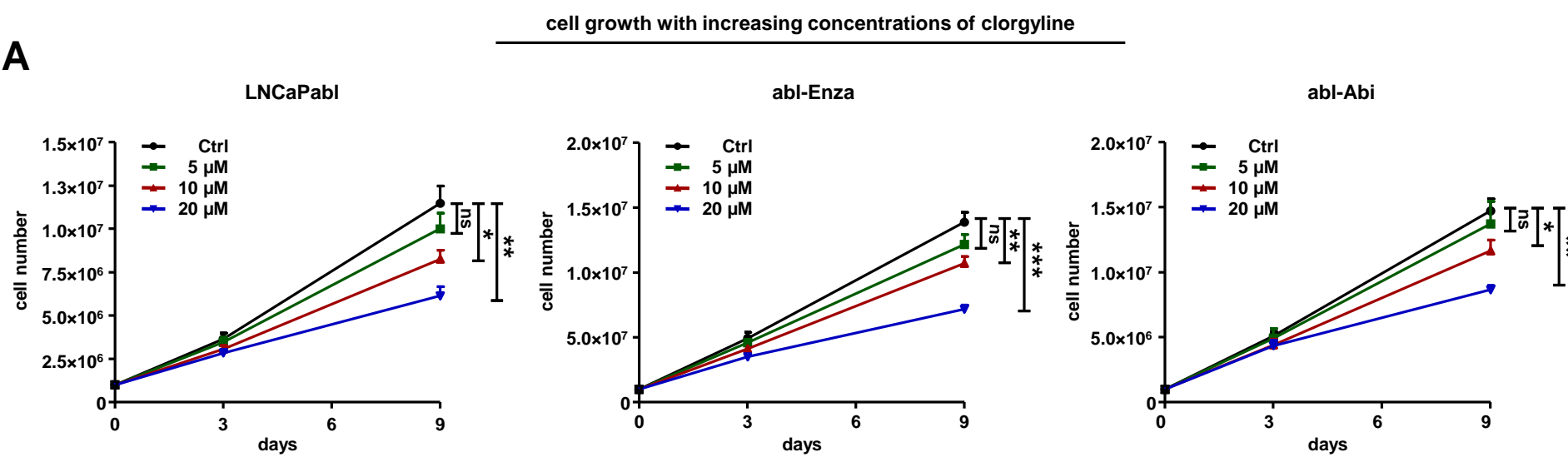

B

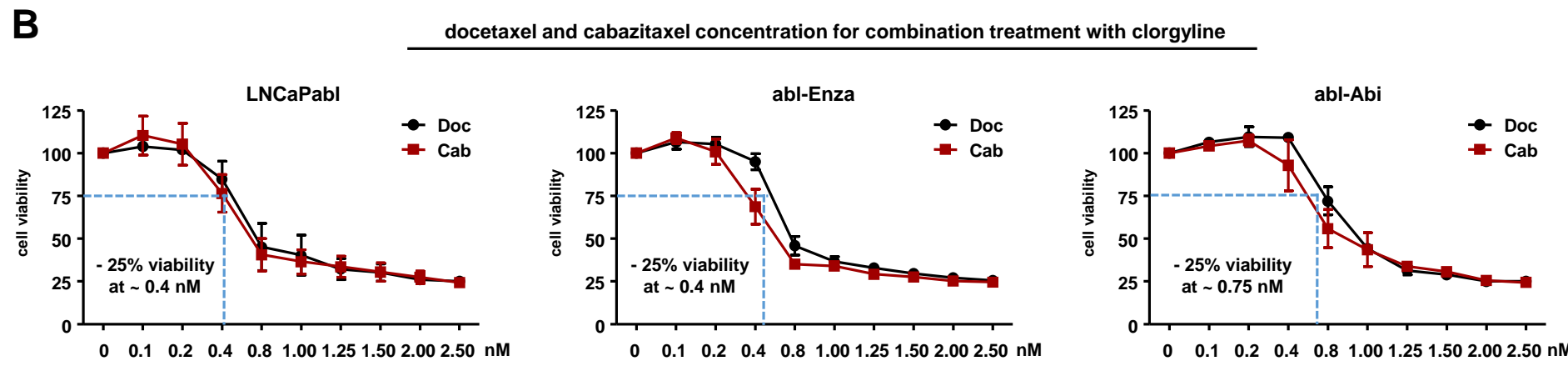

Supplement: Supplementary file 9 — Figure S9 [file 41388_2021_1754_MOESM9_ESM.pdf]
