# Supplementary material for: Targeting the glucocorticoid receptor signature gene Mono Amine Oxidase-A enhances the efficacy of chemo- and anti-androgen therapy in advanced prostate cancer
Source: Oncogene. 2021 Apr 1;40(17):3087–100. doi: 10.1038/s41388-021-01754-0 (PMC8084733; doi:10.1038/s41388-021-01754-0)
Supplement: Supplementary file 6 — Figure S6 [file 41388_2021_1754_MOESM6_ESM.pdf]

Figure S6

A

Single cell RNAseq screen for basal MAO-A mRNA expression

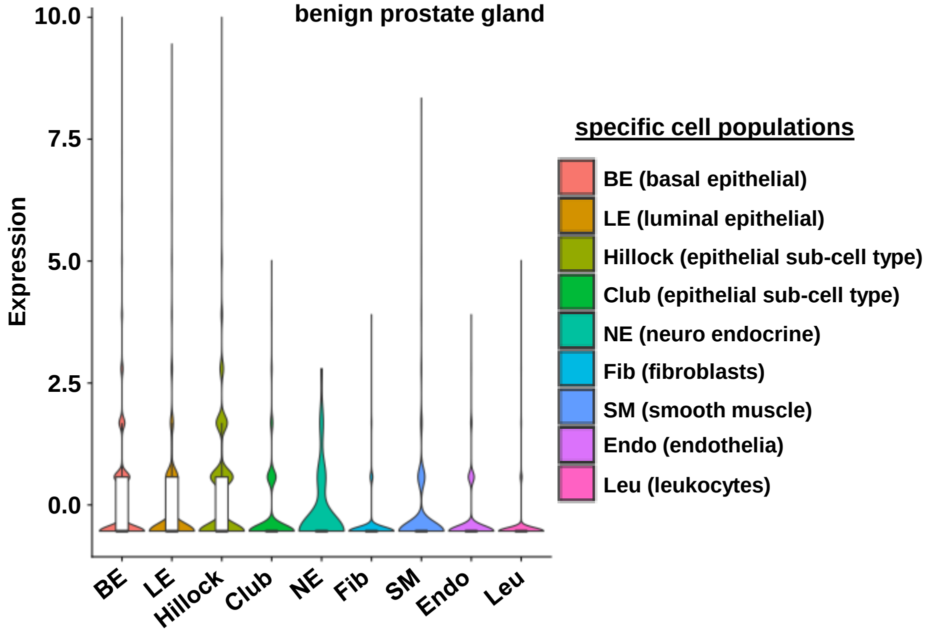

B

Oncomine data base screen

MAO-A expression
(all listed tumor entities)

CA vs. BE (MAO-A expression)	up-regulated	down-regulated
Bladder Cancer	1*(12)	1*(12)
Brain/CNS Cancer	1*(29)	2*(29)
Breast Cancer	---	30*(53)
Cervical Cancer	1*(7)	---
Colorectal Cancer	---	22*(35)
Esophageal Cancer	---	3*(11)
Gastric Cancer	---	7*(23)
Head/Neck Cancer	1*(31)	3*(31)
Kidney Cancer	---	6*(20)
Leukemia	3*(29)	---
Liver Cancer	---	6*(10)
Lung Cancer	---	18*(21)
Lymphoma	2*(35)	1*(35)
Melanoma	---	3*(5)
Myeloma	---	---
Other Cancer	1*(32)	6*(32)
Ovarian Cancer	---	6*(13)
Pancreatic Cancer	---	---
Prostate Cancer	4*(19)	---
Sarcoma	---	12*(20)
significant analyses	14	126
included analyses	457	

* n significant analyses (n total analyses)

C

significantly altered MAO-A PCa Oncomine datasets

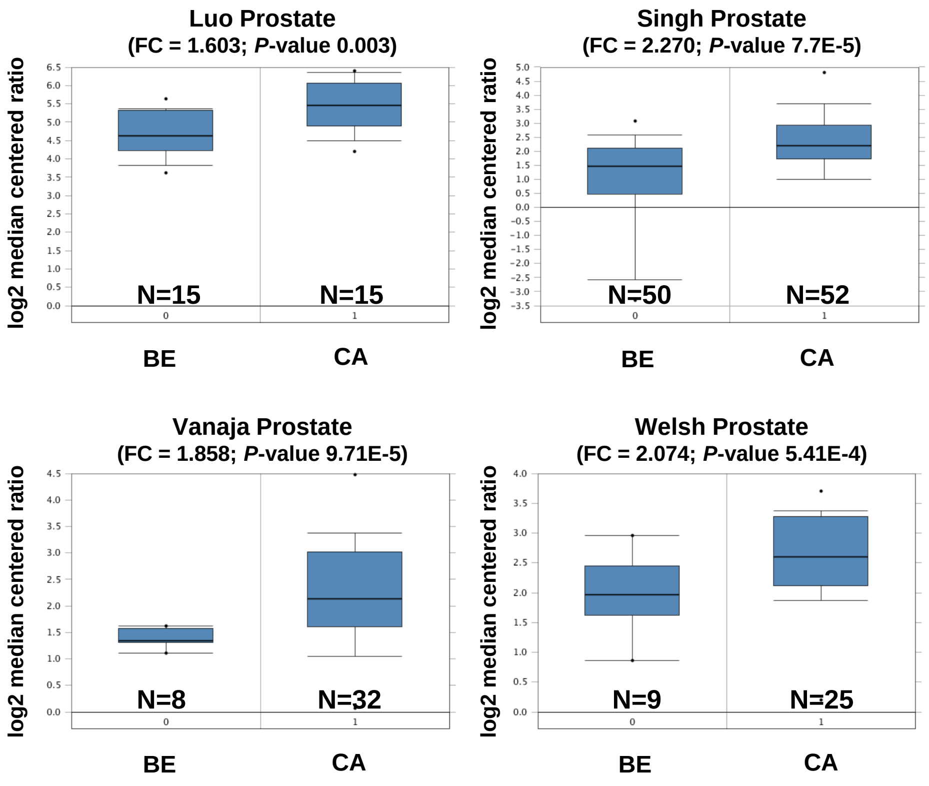

Thresholds: *P*-value: 0.01, Fold change: 1.5, Gene rank: Top10%, data type all
