# Supplementary material for: Targeting the glucocorticoid receptor signature gene Mono Amine Oxidase-A enhances the efficacy of chemo- and anti-androgen therapy in advanced prostate cancer
Source: Oncogene. 2021 Apr 1;40(17):3087–100. doi: 10.1038/s41388-021-01754-0 (PMC8084733; doi:10.1038/s41388-021-01754-0)
Supplement: Supplementary file 7 — Figure S7 [file 41388_2021_1754_MOESM7_ESM.pdf]

Figure S7

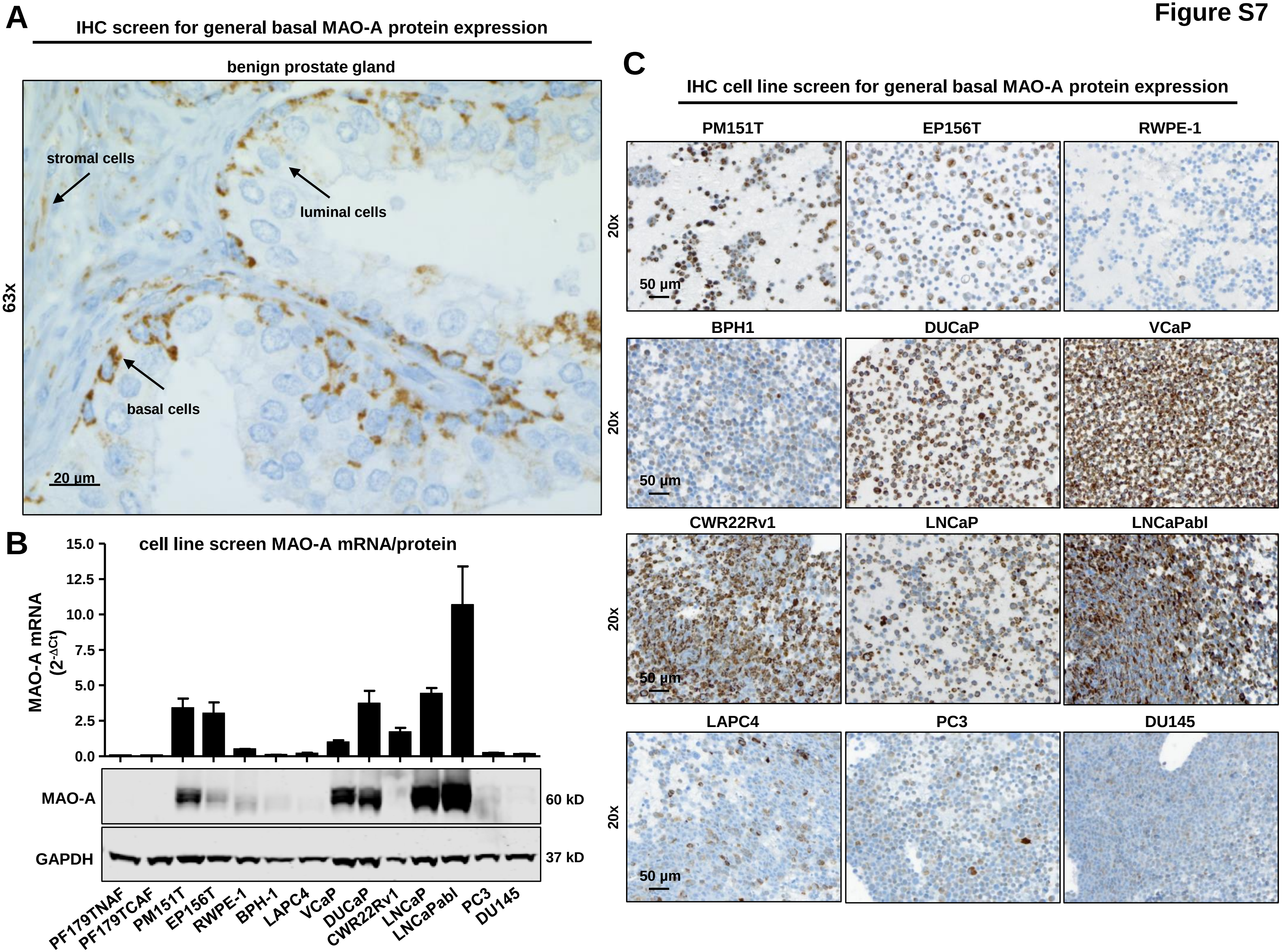

A
IHC screen for general basal MAO-A protein expression
benign prostate gland
63x
stromal cells
luminal cells
basal cells
20 µm
B
cell line screen MAO-A mRNA/protein
MAO-A mRNA
(2-ΔCt)
15.0
12.5
10.0
7.5
5.0
2.5
0.0
MAO-A
60 kD
GAPDH
37 kD
PF179TNAF
PF179TCAF
PM151T
EP156T
RWPE-1
BPH-1
LAPC4
VCaP
DUCaP
CWR22Rv1
LNCaP
LNCaPabl
PC3
DU145
C
IHC cell line screen for general basal MAO-A protein expression
PM151T
EP156T
RWPE-1
BPH1
DUCaP
VCaP
CWR22Rv1
LNCaP
LNCaPabl
LAPC4
PC3
DU145
20x
50 µm
