# Supplementary material for: Targeting the glucocorticoid receptor signature gene Mono Amine Oxidase-A enhances the efficacy of chemo- and anti-androgen therapy in advanced prostate cancer
Source: Oncogene. 2021 Apr 1;40(17):3087–100. doi: 10.1038/s41388-021-01754-0 (PMC8084733; doi:10.1038/s41388-021-01754-0)
Supplement: Supplementary file 10 — Additional File 1 [file 41388_2021_1754_MOESM10_ESM.docx]

**Additional File 1: Supplementary Material and Methods**

**Chemicals:** The following chemicals were used with concentrations as indicated in the result section and figure legends: docetaxel (Doc), cabazitaxel (Cab), clorgyline (Clor) (THP Medical Products, Vienna, Austria), dexamethasone (Dex), prednisolone (Pred), RU486, apalutamide (Apa) (Selleck Chemicals, Munich, Germany), enzalutamide (Enza), abiraterone (Abi) (Hycultech, Beutelsbach, Germany), darolutamide (Daro) (MedChemExpress, Stockholm, Sweden), dihydrotestosterone (DHT), dimethylsulfoxid (DMSO), and doxycycline (Dox) (Sigma Aldrich, Vienna, Austria).

***Ex vivo* tissue cultures:** The use of primary material was approved by the Ethics Committee of the Medical University of Innsbruck (Study no. UN4837:317/4.7). Written consent was obtained from all patients and documented in the database of the University Hospital Innsbruck in agreement with statutory provisions. Benign and cancerous tissue samples were taken from the explanted prostates by a pathologist, and further processed after positive quality control as previously described (1). Tissue samples were cultured in the presence of 100 nM Dex or DMSO for 3 days, harvested, and further processed for qRT-PCR or IHC analysis.

**Generation of lentiviral vectors & plasmid construction:** Construction of a Dox inducible short hairpin RNA (shRNA) vector against human GR (shGR-1) was done as already described (2). GR knockdown in the established PF179TCAF-shGR-1 cell sub-line was achieved by adding 1 µg/ml Dox for 48 h. The lentiviral vector for constitutive MAO-A expression with a puromycine resistance gene was generated by PCR amplification of human MAO-A cDNA (nm_000240) using TATAggcgcgccgccaccATGGAGAATCAAGAGAAGGC (AscI-Kozak-MAO-A sense (underlined)) and TATAagatcTCAAGACCGTGGCAGGAGCTTG (BglII-MAO-A antisense (underlined)) oligonucleotides. Purified MAO-A cDNA was digested with AscI and BglII and sub-cloned into the AscI/BamHI site of the lentiviral vector pHR-SFFV-TetRKRAB-iPuro, thereby exchanging TetRKRAB cDNA with the cDNA of MAO-A and generating pHR-SFFV-MAO-A-iPuro. Sequence verified plasmids were used to generate lentiviral particles. Lentiviral transduction of target cells was performed as described previously (2). 72 hours after infection, PF179TCAF, PC3, and DU145 cells were selected with 2.5 µg/ml puromycine (Sigma) for 2 weeks to obtain a clean pool of transduced cells.

**Bioinformatic analysis of microarray, RNAseq, and single cell RNAseq datasets:** 5 x 10^6^ PF179TCAF-shGR-1 cells, LNCaPabl-Abi (abl-Abi), and LNCaPabl-Enza (abl-Enza) cells were seeded into T175 tissue flasks. On the next day specific treatments were performed as followed. PF179TCAF-shGR-1: Group 1: vehicle Ctrl was treated with DMSO for 24 h. Group 2: 100 nM Dex for 24h. Group 3: cells were pretreated with 1µg/ml Dox for 48 h, followed by a 100 nM Dex and 1µg/ml Dox treatment for 24 h. Group 4: 100 nM Dex and 12 µM RU486 treatment for 24 h. Epithelial cells: Group 1: vehicle Ctrl was treated with DMSO for 24 h. Group 2: 100 nM Dex for 24h. After 24 h cells were harvested and total RNA was isolated with the Blirt EXTRACTME TOTAL RNA KIT (LabConsulting). All array experiments were performed in triplicates. Total RNA was isolated and Affymetrix microarray analyses (Clariom^TM^ S human arrays for stromal cells and Clariom^TM^ D human arrays for epithelial cells) were performed by a commercial service provider (Eurofins/Aros, Aarhus, Denmark) according to standard procedures. The experimental data have been deposited in the NBCI Gene Expression Omnibus (GEO) (GSE150432, GSE150437). Analyses were performed on gene level (filtered for Refseq mRNA annotation), differentially expressed genes were identified using moderated t-tests (R package *limma*), and p-values were adjusted for multiple testing based on the false discovery rate according to the Benjamini-Hochberg procedure. Genes with more than two-fold change at a FDR<0.1 (or >1.5 fold change and p<0.01 where stated) were considered as significantly differentially expressed. Gene set enrichment analysis on log2 fold change pre-ranked data was performed using GSEA software and gene sets for biological hallmark processes and pathways (MSigDB) as well as defined GR and AR target gene signatures. Heatmaps were generated using Genesis 1.8.1. Data were also compared to significantly upregulated genes after GC stimulation of LREX´ cells (reanalyzed data from GSE51871) (3) using Venn diagrams (<http://icbi.at/venn>).

Bioinformatic analysis of the publicly available TCGA-PRAD (4) and SU2C-PRAD (5) datasets was performed with R (v 4.0.0). The GR and AR selective target gene sets have been published previously (3) and gene set activity scores were calculated with the GSVA R package.

MAO-A mRNA expression in benign and cancerous samples of the TCGA-PRAD dataset was analyzed with the DESeq2 R package. To identify correlated pathways in the TCGA and SU2C datasets all genes were ranked based on their correlation to MAO-A expression via the formula (-log(correlation.p.value)*sign(correlation.coefficient). Pathway analysis was performed against the MSigDB hallmark gene sets with fGSEA. Single cell RNAseq data from normal and BPH human prostates were used to characterize cell type specific MAO-A mRNA expression (6, 7).

**Screening of GR binding sites:** For the identification of potential GR binding sides near the MAO-A gene, GR binding elements were extracted using publicly available ChIP-seq datasets GSE79431, GSE79803, GSE85343, GSE39879 and GSE51497 from the GEO database (3, 8-10) and visualized with the IGV browser.

**Chromatin Immunoprecipitation (ChIP):** 4 x 10^6^ PF179TCAF cells were seeded in 15 cm^2^ cell culture dishes and grown for 48 h. Specific treatment with DMSO, 100 nM Dex, or 100 nM Dex and 6 µM RU-486 was performed for 16 hours. Chromatin IP was performed with the SimpleChIP® Plus Enzymatic Chromatin Kit (Magnetic Beads) (Cell Signaling) according to the manufacturer´s protocol. Positive control Histone H3 (D2B12) XP® rabbit mAb (Cell Signaling), negative control normal rabbit IgG (Cell Signaling) or GR (D6H2L) XP® Rabbit mAb (Cell Signaling) were added to the IP samples and incubated for 16 hours at 4°C. Elution of chromatin from antibody/protein G magnetic beads as well as reversal of cross links was done according to the kit protocol. Quantification of DNA was done by qRT-PCR on an ABI PRISM 7500-FAST system (ThermoFisher Scientific) using specific primers and probes for 2 identified GR MAO-A binding sides: ChIP-MAO-A R1-fwd, 5´-AGC TTA CTT GAG TCC TAA CCG T-3´; ChIP-MAO-A R1-rev, 5´-AAG GGC TTC TCA TTG TCA GC-3´; ChIP-MAO-A R1-probe, 5´-GAA AAG GGA GGG GTA GTC AGA-3´; ChIP-MAO-A R2-fwd, 5´-GTG TTT TGG GGC ACG GTT C-3´; ChIP-MAO-A R2-rev, 5´-CTA AGA GGG TGA AGC AGG GG-3´; ChIP-MAO-A R2-probe, 5´-GGA CAT TGC GTT CTG CTT GA-3´) and for the internal positive control Beta-2-microglobulin (B2M), ChIP-B2M fwd, 5´-TGC TGT CTC CAT GTT TGA TGT ATC T-3´; ChIP-B2M-rev, 5´-TCT CTG CTC CCC ACC TCT AAG T-3´; ChIP-B2M-probe, 5´-CAG GTT GCT CCA CAG GTA GCT CTA G-3´.

**RNA isolation from prostate cryo-tissue:** 40 representative RPE tissues were selected from the Innsbruck database as described above. Tissue processing and total RNA isolation was performed as previously described (2).

**RNA isolation from ex vivo tissue culture material and cell lines:** Tissue samples were homogenized with 5-mm steel milling balls using a TissueLyser II (Qiagen) at 30 Hz for 2 minutes prior to RNA isolation. Total RNA from tissue and cell lines was isolated with the Blirt EXTRACTME TOTAL RNA KIT (LabConsulting, Vienna, Austria) according to the manufacturer's instructions. RNA yield and quality were determined on a Nanodrop2000 system (ThermoFisher Scientific, Vienna, Austria).

**Proliferation & viability measurements:** Proliferation was assessed using [^3^H] thymidine incorporation. Cell viability was determined using the CellTiter 96® AQueous one solution assay (Promega) according to the manufacturer`s protocol. Briefly, 1.500 cells/96-well PC3, DU145, 2.500 cells/96-well PF179TCAF, 10.000 cells/96-well LNCaP, LNCaPabl, abl-Enza, and abl-Abi were seeded in 96-well plates and incubated for specific time points. Treatment with the indicated drugs was performed on the next day. Measurements were done in at least 3 independent biological experiments with at least 3 technical replicates.

For MAO-A knockdown experiments, cells were transfected twice in a period of 9 days using either 25 nM ON-TARGETplus Human MAO-A siRNA SMARTpool (L-009369-00-0005), or siControl On-Target plus Non-targeting pool (D-001810-10-05) and Lipofectamine2000 (ThermoFisher Scientific) according to the manufacturer`s protocols.

**Cell growth analysis & Counting:** For single and combination treatments 1 x 10^6^ LNCaP, LNCaPabl, abl-Abi, and abl-Enza cells were seeded in T25 flasks, treated with specific drugs, and incubated for 3 days. At day 3, cell numbers were determined by a CASY cell counter (Schärfe System, Reutlingen, Germany). 8 x 10^5^ cells were re-seeded, and treated again with specific drugs for additional 3 days. At day 6, medium and drugs were changed. At day 9, cells were finally counted and further processed for different approaches.

**Apoptosis & cell cycle distribution & CD24/CD44 measurement:** The percentage of apoptotic cells as well as cell cycle distribution have been determined using FACS Calibur (Becton Dickinson, Heidelberg, Germany). Identification and specific labeling of CD24^low^-CD44^high^ cell sub-population was performed as previously described (11).

**References**

1. Handle F, Puhr M, Schaefer G, Lorito N, Hoefer J, Gruber M, et al. The STAT3 Inhibitor Galiellalactone Reduces IL6-Mediated AR Activity in Benign and Malignant Prostate Models. Mol Cancer Ther. 2018;17(12):2722-31.

2. Puhr M, Hoefer J, Eigentler A, Ploner C, Handle F, Schaefer G, et al. The Glucocorticoid Receptor Is a Key Player for Prostate Cancer Cell Survival and a Target for Improved Antiandrogen Therapy. Clin Cancer Res. 2018;24(4):927-38.

3. Arora VK, Schenkein E, Murali R, Subudhi SK, Wongvipat J, Balbas MD, et al. Glucocorticoid receptor confers resistance to antiandrogens by bypassing androgen receptor blockade. Cell. 2013;155(6):1309-22.

4. Cancer Genome Atlas Research N. The Molecular Taxonomy of Primary Prostate Cancer. Cell. 2015;163(4):1011-25.

5. Abida W, Cyrta J, Heller G, Prandi D, Armenia J, Coleman I, et al. Genomic correlates of clinical outcome in advanced prostate cancer. Proc Natl Acad Sci U S A. 2019;116(23):11428-36.

6. Henry GH, Malewska A, Joseph DB, Malladi VS, Lee J, Torrealba J, et al. A Cellular Anatomy of the Normal Adult Human Prostate and Prostatic Urethra. Cell Rep. 2018;25(12):3530-42 e5.

7. Joseph DB, Henry GH, Malewska A, Iqbal NS, Ruetten HM, Turco AE, et al. Urethral luminal epithelia are castration-insensitive cells of the proximal prostate. Prostate. 2020;80(11):872-84.

8. Kadiyala V, Sasse SK, Altonsy MO, Berman R, Chu HW, Phang TL, et al. Cistrome-based Cooperation between Airway Epithelial Glucocorticoid Receptor and NF-kappaB Orchestrates Anti-inflammatory Effects. J Biol Chem. 2016;291(24):12673-87.

9. Nakamoto M, Ishihara K, Watanabe T, Hirosue A, Hino S, Shinohara M, et al. The Glucocorticoid Receptor Regulates the ANGPTL4 Gene in a CTCF-Mediated Chromatin Context in Human Hepatic Cells. PLoS One. 2017;12(1):e0169225.

10. Sahu B, Laakso M, Pihlajamaa P, Ovaska K, Sinielnikov I, Hautaniemi S, et al. FoxA1 specifies unique androgen and glucocorticoid receptor binding events in prostate cancer cells. Cancer Res. 2013;73(5):1570-80.

11. Puhr M, Hoefer J, Schafer G, Erb HH, Oh SJ, Klocker H, et al. Epithelial-to-mesenchymal transition leads to docetaxel resistance in prostate cancer and is mediated by reduced expression of miR-200c and miR-205. Am J Pathol. 2012;181(6):2188-201.
