# Supplementary material for: Targeting the glucocorticoid receptor signature gene Mono Amine Oxidase-A enhances the efficacy of chemo- and anti-androgen therapy in advanced prostate cancer
Source: Oncogene. 2021 Apr 1;40(17):3087–100. doi: 10.1038/s41388-021-01754-0 (PMC8084733; doi:10.1038/s41388-021-01754-0)
Supplement: Supplementary file 11 — Aditional File 2 Table S1 [file 41388_2021_1754_MOESM11_ESM.pdf]

Additional File 2 Table S1:

stromal and epithelial data set comparisons. Significantly up-regulated genes (>1.5 FC FDR <0.1) between different datasets. These data are the basis for Figure 1 E, F

4	23	1	78	20	159	9
FKBP5	SGK1	PER1	SAA2	PGC	TUBA3C	PTGER4
MAOA	KLK4		SAA1	FAXDC2	TUBA3E	CRISPLD2
ERRFI1	SPSB1		IRS2	COLCA1	PGLYRP2	ELL2
KLF9	HTR3A		CXCL8	MYBPC1	C5orf4	EEF2K
	SCNN1G		FLVCR2	KLK2	SLC25A18	NFKBIA
	C19orf48		METTL7A	MAP1B	SNAI2	TSC22D3
	RGS2		CPM	CST1	EMP1	FOXO3
	TUBA3D		SLC7A2	IL6R	RASD1	MT1X
	TMPRSS2		AOX1	FAM43A	SLC45A3	IFNGR1
	RHOU		CXCL1	DUSP4	TFAP2A	
	ETS2		ADH1B	IGF1R	BIRC3	
	MBOAT2		IFI44L	LCP1	S100P	
	NPC1		ITGA10	PLPP1	GADD45B	
	ZC3H12A		FAM107A	TMPRSS11E	TRIM36	
	STK39		PTK2B	BBS12	CEBPD	
	FAM105A		GCNT1	TMCC3	KCNMA1	
	FAM49A		DUSP5	AKAP5	PSD	
	CHKA		PISD	HSD17B11	TRIM48	
	HOMER2		FAM167A	GALNT2	KLF5	
	FBXO38		PXYLP1	CFAP69	KLF15	
	PRKCD		DNAJB4		C9orf152	
	TBC1D8		PPME1		DBC1	
	TPM1		LRRC16A		ALDH1A3	
			SORT1		HERC5	
			ZBTB16		SLC25A27	
			TRNP1		PPP1R1B	
			FABP4		GSG1	
			PARM1		ACTA2	
			TCEAL4		C11orf92	
			PRKAG2		PRR15L	
			ABLIM3		PMEPA1	
			SLC44A1		FHL2	
			PKDCC		PHLDA1	
			ARMC8		ADAMTSL4	
			SLC7A8		EDG7	
			KRT10		LEPREL1	
			GGT5		STX19	

			CXCL6		C7orf63	
			CHCHD7		AHNAK	
			SP110		RND3	
			FPR1		NKX3-1	
			AGL		MICAL2	
			CLDN7		KIAA0040	
			C1QL1		GREB1	
			TCEAL3		C17orf48	
			ATOH8		LPAR3	
			ITGA5		ZMIZ1	
			DPY19L4		PDE9A	
			YEATS2		F2RL1	
			SLC16A12		ABHD2	
			RASL11B		MIB1	
			ELF1		C1orf149	
			TCAF2		FLJ30428	
			DIAPH2		CA13	
			ITPR1		ECAT8	
			GK5		SPIC	
			TCEAL1		CRY2	
			SLC26A6		TSKU	
			NID2		KRT80	
			SRPX2		TSPYL2	
			ALOX5AP		HMOX2	
			EPS8		RAB3B	
			ANXA4		RHOB	
			TCEAL6		NEDD4L	
			SSB		HSD11B2	
			ITPK1		PPAP2A	
			WBP5		NDRG1	
			AKAP13		MEAF6	
			FAM222B		STEAP2	
			TBC1D12		SLC2A12	
			TNIP1		AGXT2L1	
			ARRDC1		KBTBD11	
			GALNT15		PCDH10	
			PCYT2		LIN7B	
			MT1F		GPR125	
			MORF4L2		ARG1	
			PTPN1		GHR	
			ZHX3		SLC31A2	
					BRDT	
					SPRYD5	
					KCNRG	
					TMEM164	

					ABCC4	
					C1orf116	
					DDIT4	
					HERC6	
					PPFIBP1	
					DOCK5	
					PDCD11	
					DNAJB5	
					FRMD3	
					TBC1D4	
					KIF5C	
					FLJ22795	
					STAT3	
					PER2	
					C17orf58	
					BCL6	
					GADD45G	
					CGNL1	
					NRP1	
					MTMR9	
					SIPA1L2	
					CA12	
					C6orf85	
					STEAP1	
					ATAD2	
					OFD1	
					FRYL	
					PACSIN2	
					RRAS	
					TCP10L	
					AXUD1	
					SH3BGR	
					KIAA1370	
					PLLP	
					SOCS2	
					KLF6	
					RDH10	
					ZBTB25	
					RAB20	
					CYR61	
					MLPH	
					LOC652968	
					ZYX	
					IL1R1	
					ARHGAP28	

					PNPLA8	
					SOX13	
					NCOA6IP	
					PLXNA1	
					TNFAIP8	
					GABRB3	
					RBM24	
					DUSP1	
					AR	
					TNFRSF10B	
					REPS2	
					PPFIBP2	
					LOC643896	
					MED23	
					TUBB2A	
					ALAD	
					TEF	
					EHF	
					MTE	
					LRRFIP2	
					DNM1L	
					GRHL2	
					SFXN2	
					RER1	
					ZC3HAV1	
					SDC1	
					ZNF622	
					FOXD4	
					GOLSYN	
					FNBP1L	
					MAP3K6	
					OSR2	

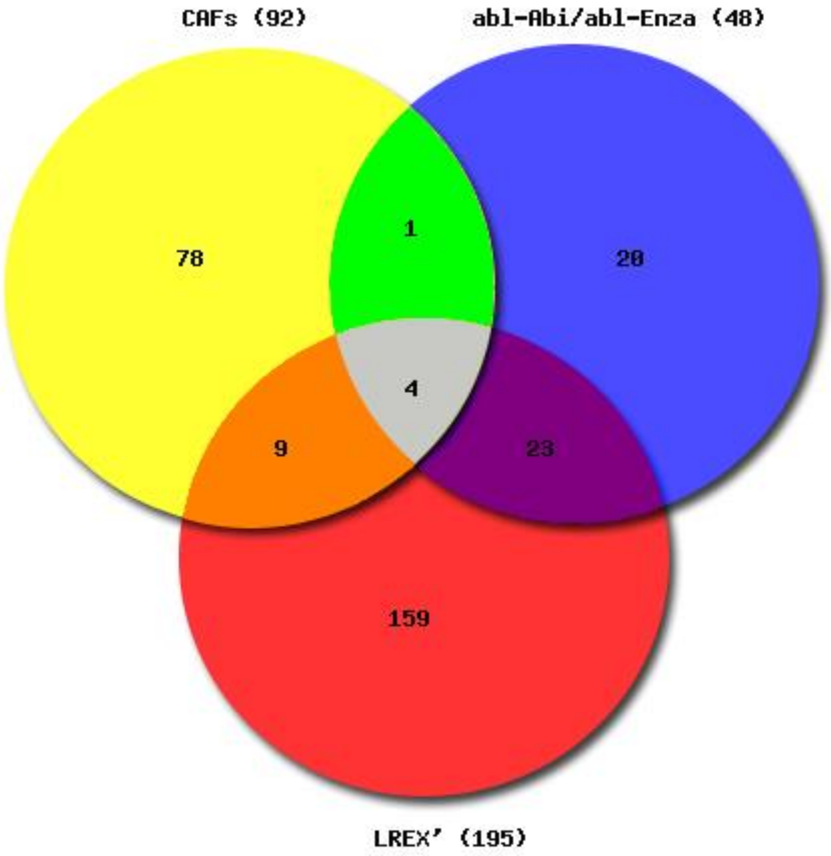

4	mutual genes CAF, LNCaPabl-Abi/Enza, LREX´
23	mutual genes , LNCaPabl-Abi/Enza, LREX´
1	mutual genes , CAF, LNCaPabl-Abi/Enza, LREX´
78	genes CAF
20	genes LNCaPabl-Abi/Enza
159	genes LREX´
9	mutual genes CAF, LREX´
