# Supplementary material for: Targeting the glucocorticoid receptor signature gene Mono Amine Oxidase-A enhances the efficacy of chemo- and anti-androgen therapy in advanced prostate cancer
Source: Oncogene. 2021 Apr 1;40(17):3087–100. doi: 10.1038/s41388-021-01754-0 (PMC8084733; doi:10.1038/s41388-021-01754-0)
Supplement: Supplementary file 12 — Additional File 3 Table S2, S3 [file 41388_2021_1754_MOESM12_ESM.pdf]

Additional File 3 Table S2

Characteristics and descriptive histology at time of RPE of all included patients in the project.

**patients included within the study**<br>**(patients characteristics and descriptive histology at time of RPE)**			
**patients in total (n=202)**	**mean**	**median**	**range**
age (202/202)	62	62	41-82
PSA (ng/ml) (189/202)	5.93	4.57	1.35-54.7
free PSA% (159/202)	14.96	13.3	3.1-34.7
prostate weight (g) (191/202)	40.8	40	18-85
**GS at time of RPE (192/202)**	**numbers**	**% in total**	
GS 5–6	57	30	
GS 7	94	49	
GS 8–10	41	21	
**pT-stage at time of RPE (192/202)**	**numbers**	**% in total**	
pT2a	14	7	
pT2b	2	1	
pT2c	107	55	
pT3a	50	26	
pT3b	15	8	
pT4	4	2	
**biochemical tumor relapse**	**no**	**yes**	
patients (202/202)	131	71	
time to relapse (months) (71/202)	---	40 (mean)<br>1.6-126.9 (range)	
**tumor specific treatment before RPE**	**no**	**yes**	
patients (202/202)	202	0	

Additional File 3 Table S3

Characteristics and descriptive histology at time of RPE of included relapse patients.

**included relapse patients in total (n=65)** (patients characteristics and descriptive histology at time of RPE)			
**group 1 patients (n=34)** (patients with low-intermediate MAO-A IRS)	**mean**	**median**	**range**
age (34/34)	64	64	51-74
PSA (ng/ml) (34/34)	7.3	5.5	1.9-32.0
free PSA% (30/34)	14.5	13.3	5.4-34.1
**GS at time of RPE (34/34)**	**numbers**	**% in sub-group (n=34)**	**% in total (n=65)**
GS 5–6	1	3	2
GS 7	21	62	32
GS 8–10	12	35	18
**pT-stage at time of RPE (34/34)**	**numbers**	**% in sub-group (n=34)**	**% in total (n=65)**
pT2a	2	6	3
pT2b	2	6	3
pT2c	11	32	17
pT3a	16	47	25
pT3b	3	9	5
pT4	0	0	0
**group 2 patients (n=31)** (patients with strong MAO-A IRS)	**mean**	**median**	**range**
age (31/31)	63	64	41-76
PSA (ng/ml) (31/31)	7.0	5.0	1.9-17.2
free PSA% (27/31)	17.5	15.5	6.8-37.3
**GS at time of RPE (31/31)**	**numbers**	**% in sub-group (n=31)**	**% in total (n=65)**
GS 5–6	2	6	3
GS 7	14	45	22
GS 8–10	15	48	23
**pT-stage at time of RPE (31/31)**	**numbers**	**% in sub-group (n=31)**	**% in total (n=65)**
pT2a	2	6	3
pT2b	0	0	0
pT2c	7	23	11
pT3a	12	39	18
pT3b	8	26	12
pT4	2	6	3
