# Supplementary material for: Targeting the glucocorticoid receptor signature gene Mono Amine Oxidase-A enhances the efficacy of chemo- and anti-androgen therapy in advanced prostate cancer
Source: Oncogene. 2021 Apr 1;40(17):3087–100. doi: 10.1038/s41388-021-01754-0 (PMC8084733; doi:10.1038/s41388-021-01754-0)
Supplement: Supplementary file 13 — Supplementary information [file 41388_2021_1754_MOESM13_ESM.docx]

**Supplementary information:**

**additional file 1.** Supplementary material and methods.

**additional file 2.**

**table S1.** Stromal and epithelial data set comparisons. Mutual significantly up-regulated genes (>1.5 FC *FDR* <0.1) between different datasets.

**additional file 3.**

**table S2.** Characteristics and descriptive histology at time of RPE of all included patients in the project.

**table S3.** Characteristics and descriptive histology at time of RPE of included relapse patients.

**Supplementary Figure Legends:**

**Figure S1**: Gene enrichment signatures for GR and AR signaling. **(A, B)** Gene-set enrichment blots for a significantly elevated GR and AR signature after 100 nM Dex treatment in stromal PF179TCAF-shGR-1 datasets as well as in epithelial abl-Enza, abl-Abi and the fused abl-Enza/Abi datasets after treatment with 100 nM Dex for 24 h. **(C)** Internal GR-ChIP quality control for GR antibody specificity with beta-2 microglobulin (B2MG). Expression of B2MG is only detected in the 2% input fraction and in the H3 fraction but not in the GR and IgG fraction.

**Figure S2:** MAO-A antibody specificity testing. **(A)** Western blot analysis reveals a single MAO-A band at approximately 55kD after 3 d treatment with 100 nM Dex using PF179TCAF cells. **(B)** MAO-A mRNA and protein expression after 3 d treatment with 100 nM Dex alone or in combination with 6 µM RU486 of PF179TCAF cells. **(C)** MAO-A protein expression of PC3 cells containing a MAO-A constitutive overexpression or control vector. **(D)** Reduced MAO-A protein expression after 3 d transient MAO-A knockdown with specific siMAO-A (25 nM, 50 nM) in LNCaPabl cells. **(E)** MAO-A IHC staining of PF179TCAF cells after 3 d treatment with 100 nM Dex alone or in combination with 6 µM RU486. Magnification: 20x (scale bar = 50 µm). **(F)** Quantification of MAO-A protein expression in abl-Enza, abl-Abi, PF179TCAF, PC3, DU145, and CWR22Rv1 cells after 100 nM Dex treatment for 3 days (unpaired t-test; *, *P*< 0.05; **, *P*< 0.01; ***, *P*< 0.001).

**Figure S3:** MAO-A expression in benign tissues after neoadjuvant chemotherapy. **(A)** Quantification of epithelial and stromal MAO-A IRS within benign areas of 13 selected chemotherapy and control patients (Mann-Whitney test; *, *P*< 0.05, ***, *P*< 0.001; Box Whisker Plot with 10-90 percentile) and representative microscopy images. Magnification: 20x (scale bar = 50 µm) and 63x (scale bar = 20 µm). **(B)** Elevated MAO-A protein expression in generated constitutive MAO-A overexpressing PF179TCAF, PC3 and DU145 cell sub-lines compared to corresponding control lines. **(C)** Representative pictures for possible altered cell morphology of constitutive MAO-A overexpressing PF179TCAF, PC3, and DU145 cell sub-lines compared to corresponding control lines.

**Figure S4:** MAO-A overexpression and EMT. **(A)** qRT-PCR screen for altered mRNA expression of diverse EMT markers in MAO-A overexpressing compared to control PC3 and DU145 cell sub-lines. Data represent mean + SE from 3 independent experiments (unpaired t-test; *, *P*< 0.05; **, *P*< 0.01; ***, *P*< 0.001). **(B)** Representative Western blot bands and quantification of E-cadherin and Vimentin protein in MAO-A overexpressing compared to control PC3 and DU145 cell sub-lines. Data represent mean + SE from 3 independent experiments (unpaired t-test; *, *P*< 0.05; ***, *P*< 0.001). **(C)** qRT-PCR screen for altered mRNA expression of diverse EMT markers and representative Western blot bands for E-cadherin and Vimentin protein after transient MAO-A knockdown using 25 nM specific siMAO-A for 3 d in PC3 and DU145 cells mRNA data represent mean + SE from 3 independent experiments (unpaired t-test; *, *P*< 0.05; ***, *P*< 0.001).

**Figure S5:** MAO-A overexpression and stemness. **(A)** qRT-PCR screen for altered mRNA expression of diverse stem cell markers in MAO-A overexpressing compared to control PC3 and DU145 cell sub-lines. Data represent mean + SE from 3 independent experiments (unpaired t-test; *, *P*< 0.05; **, *P*< 0.01; ***, *P*< 0.001). **(B)** CD24 and CD44 antibody specificity testing for FACS analysis in PC3 cells. **(C)** Measurement of CD44 protein expression of MAO-A overexpressing compared to control PC3 and DU145 cell sub-lines. **(D)** Representative FACS analysis pictures and quantification of the CD24^low^-CD44^high^ cell sub-population in MAO-A overexpressing compared to control PC3 and DU145 cell sub-lines. (unpaired t-test; **, *P*< 0.01).

**Figure S6:** General MAO-A mRNA expression. **(A)** Single cell RNAseq cell type specific MAO-A mRNA expression in normal and BPH human prostate tissue. **(B)** Analysis of MAO-A gene expression in different malignancies in the publicly available Oncomine database (threshold *P*-value 0.01, threshold fold change 1.5, top 10%, data type all). **(C)** For PCa, the *Luo-Prostate*, *Singh-Prostate*, *Vanja-Prostate*, and *Welsh-Prostate* datasets showed significantly elevated MAO-A gene expression in cancerous tissues. No analyses resulted in significantly reduced MAO-A gene expression in PCa datasets (threshold *P*-value 0.01, threshold fold change 1.5, top 10%, data type all).

**Figure S7:** Cell specific MAO-A mRNA and protein expression. **(A)** Representative high-resolution IHC picture of a benign prostate gland for MAO-A protein staining. Magnification: 63x (scale bar = 20 µm). **(B)** Prostate cell line screen for basal MAO-A mRNA and protein expression. **(C)** Representative pictures from an IHC prostate cell line screen for basal MAO-A protein expression. Magnification: 20x (scale bar = 50 µm).

**Figure S8:** Correlation of MAO-A expression with AR-GR activity and GSEA pathway analysis. **(A)** MAO-A is significantly positively correlated with AR activity within the public available TCGA-PRAD and SU2C-PRAD datasets. **(B)** GSEA pathway analysis of the TCGA-PRAD and SU2C-PRAD datasets showing the correlation of MAO-A expression with the MSigDB Hallmark gene-sets. All pathways with a *FDR* < 0.05 in both datasets are shown. **(C)** Identification of identical GR and AR binding sites (R1 and R2) near the MAO-A gene in LREX´, LNCaP-F15, and VCaP cells, screening publicly available ChIP-seq datasets. **(D)** Significantly reduced cell viability and cell proliferation after 9 d siMAO-A knockdown in LNCaPabl, abl-Enza, and abl-Abi cells. (unpaired t-test; *, *P*< 0.05; **, *P*< 0.01; ***, *P*< 0.001)

**Figure S9:** Concentration testing for clorgyline and docetaxel. **(A)** Measurement of LNCaPabl, abl-Enza, and abl-Abi cell growth after 9 d treatment with increasing concentrations (5 µM, 10 µM, 20 µM) of the specific MAO-A inhibitor clorgyline. Data represent mean + SE from at least 3 independent experiments (one-way ANOVA and correction for multiple testing using Bonferroni´s comparison test; *, *P*< 0.05; **, *P*< 0.01; ***, *P*< 0.001). **(B)** Measurement of LNCaPabl, abl-Enza, and abl-Abi cell viability after 5 d treatment with increasing concentrations of docetaxel or cabazitaxel.
